# The prognostic nutritional index vs. MELD for short-term mortality in critically ill liver disease: a retrospective multi-cohort study

**DOI:** 10.3389/fnut.2026.1930417

**Published:** 2026-09-07

**Authors:** Lujun Shao, Yue Li, Cheng Xu, Li Wang, Huiyu Tai

**Affiliations:** 1 Department of Intensive Care Unit, The Affiliated Taizhou People's Hospital of Nanjing Medical University, Taizhou, China; 2 Graduate School of Medicine, Hirosaki University, Hirosaki, Japan

**Keywords:** critical illness, liver cirrhosis, malnutrition, MELD, mortality, nutritional risk screening, prognostic nutritional index, serum albumin

## Abstract

Malnutrition, sarcopenia and frailty are common in advanced liver disease and predict poorer survival, encouraging simple blood-based indices when formal assessment is impractical. The most widely used, the Prognostic Nutritional Index (PNI), combines serum albumin and the peripheral lymphocyte count; yet both have been reported to be strongly influenced by systemic inflammation as well as by nutrition, and the index is seldom benchmarked against a disease-specific reference or against albumin alone. We evaluated whether the PNI provides clinically useful prognostic information for short-term mortality in critically ill patients with liver disease, using three independent cohorts: a North American intensive-care primary cohort (*n* = 3,653), a multicentre external-validation cohort (*n* = 922) and a single-center Chinese cohort in which hepatitis B was the leading cause of liver disease (*n* = 453). In each cohort we quantified discrimination of 28-day or in-hospital mortality, compared the PNI with serum albumin alone and with the Model for End-Stage Liver Disease (MELD), and tested whether it improved discrimination or reclassification beyond MELD. The PNI discriminated death only marginally better than chance (area under the curve 0.52–0.56) and no better than albumin alone, so its lymphocyte component added no independent signal; the weak per-standard-deviation association seen in the primary cohort was not significant in either validation cohort. MELD discriminated substantially better everywhere (0.67–0.70). The conventional PNI threshold classified 89–97% of patients as abnormal and was uninformative. Across three case-mixes the PNI behaved as a weak, non-specific prognostic discriminator, adding nothing to MELD, MELD-Na or MELD 3.0; in an exploratory paired subgroup, re-measurement on days 3–7 did not improve discrimination. This is consistent with its components tracking systemic inflammation and chronic physiologic reserve—an axis largely orthogonal to the acute organ failure that drives short-term mortality. Separating the index into its components and testing incremental value provides the comparative evidence this literature has lacked. The PNI was assessed as a discriminator of short-term mortality, not as a measure of nutritional status: nutritional risk is better identified with instruments validated for that purpose, and mortality risk with MELD.

## 1 Introduction

In cirrhosis and other forms of advanced liver disease, depletion of muscle and the syndromes of malnutrition, sarcopenia and frailty are common, and each is independently associated with mortality, infection and other decompensating complications (1, 2). Reflecting this prognostic importance, hepatology and clinical-nutrition societies recommend routine nutritional-risk screening and assessment in patients with chronic liver disease (3), and consensus frameworks such as the Global Leadership Initiative on Malnutrition (GLIM) now formalize the diagnosis of disease-related malnutrition, explicitly recognizing inflammation as an aetiologic criterion alongside reduced intake and depleted reserves (4).

Comprehensive assessment of nutritional and muscle status—through body-composition imaging or performance-based frailty testing—is informative but resource-intensive, operator-dependent and often impractical in acutely or critically ill patients (5). This has motivated a search for simple surrogates derived from routine blood tests. Among the most widely adopted is the Prognostic Nutritional Index (PNI), introduced by Onodera and colleagues and defined as 10 × serum albumin (g/dL) + 0.005 × total lymphocyte count (cells/μL) (6). Conceived to gauge operative risk in patients undergoing gastrointestinal surgery, the PNI has since been reported to predict survival across a broad range of malignancies, although the supporting evidence is dominated by retrospective studies of generally low certainty (7); in the liver, a low PNI has been linked to poorer survival in hepatocellular carcinoma and chronic liver disease (8). Because it is obtained from two variables present in any routine blood panel, at no additional cost or operator burden, the index is especially appealing in acute and resource-limited settings—which makes establishing whether it genuinely adds prognostic information a question of real clinical consequence.

The prognostic meaning of the PNI in acute and critical illness is nonetheless uncertain, because both of its components are governed by factors other than nutrition. Serum albumin is a negative acute-phase reactant: during systemic inflammation its plasma concentration falls through increased transcapillary escape and accelerated degradation rather than through depleted body stores, so hypoalbuminaemia largely reflects the severity of the underlying illness and is not, in itself, an indication for nutritional therapy (9). The peripheral lymphocyte count is similarly shaped by acute stress and, in cirrhosis specifically, by cirrhosis-associated immune dysfunction—a syndrome of concurrent systemic inflammation and immune deficiency, including lymphopenia, that is driven by the liver disease itself (10). In critically ill patients with liver disease, a fixed-weight composite of albumin and lymphocytes may therefore behave more as an index of systemic inflammation and chronic physiologic reserve than of nutritional state.

For short-term mortality in advanced liver disease, the Model for End-Stage Liver Disease (MELD)—computed from serum bilirubin, creatinine and the international normalized ratio—is the established, disease-specific reference standard (11), and its sodium-containing refinement and recent recalibration (MELD 3.0) remain the benchmark for mortality prediction and organ-allocation priority (12). Whether a generic nutritional-inflammatory index contributes anything beyond this benchmark is the clinically decisive question, and answering it requires more than a demonstrated association with death: a candidate marker earns clinical value only if it improves discrimination or reclassification over established models, replicates in independent populations, and is shown not to be a redundant proxy for information already captured—principles long established for appraising novel risk markers but seldom applied to the PNI (13). Much of the PNI literature in liver disease rests on unadjusted or single-cohort associations and rarely benchmarks the index against MELD, or even against its own albumin component, leaving its incremental value largely untested.

We therefore undertook a multi-cohort appraisal of the PNI as a marker of mortality in critically ill adults with liver disease. Drawing on three independent cohorts—a North American primary cohort, a separate multi-center North American validation cohort, and a single-center Chinese cohort in which hepatitis B is the leading cause of liver disease—we asked three questions: whether the PNI discriminates death better than chance; whether it outperforms its own albumin component; and whether it adds any clinically meaningful information beyond MELD. This is not a test against a straw man: the PNI is actively promoted as a prognostic index in liver disease—including reports placing it on a par with MELD—so whether it adds information beyond MELD, or even beyond its own albumin component, is precisely what a direct multi-cohort appraisal must establish. Rather than adding one more setting in which a low PNI is associated with worse outcomes, our objective was to subject this widely used nutritional index to a consistent, disease-specific appraisal across populations, and so to provide the rigorous multi-cohort evidence needed to establish whether the PNI should inform mortality risk stratification in critically ill liver disease—or whether prognostication and nutritional risk are better anchored to tools validated for those purposes.

## 2 Materials and methods

### 2.1 Study design and data sources

This was a retrospective, multi-cohort observational study designed to evaluate the discrimination and the incremental prognostic value of the Prognostic Nutritional Index (PNI) relative to the Model for End-Stage Liver Disease (MELD) in critically ill adults with liver disease. The PNI, computed from serum albumin and the peripheral lymphocyte count, is widely promoted as a simple bedside index of nutritional and immunological status and has been reported to predict outcomes across many conditions; whether it carries genuine prognostic value in critically ill liver disease—a setting in which both of its inputs are heavily perturbed by acute illness rather than by nutrition alone—has not been rigorously tested against the disease-specific reference standard. The study used three cohorts: a **primary cohort**, an independent **external-validation cohort**, and a single-center **institutional validation cohort** assembled at the authors' center (Taizhou People's Hospital, affiliated to Nanjing Medical University, Jiangsu Province, China). The institutional cohort was included to provide validation in a non-public clinical dataset and in a population whose liver disease is more often hepatitis-B-related than alcohol-related, because discrimination findings derived solely from North American research databases may not transport to other regions and case-mixes. The study was reported in accordance with the STROBE statement (14) for observational studies; reporting of the prognostic-marker analyses was additionally informed by the TRIPOD+AI recommendations, which apply principally to the MELD + PNI incremental-model analysis (15); the completed reporting checklists are provided as separate files at submission.

The **primary cohort** was drawn from the Medical Information Mart for Intensive Care IV (MIMIC-IV) (16), a single-center database of patients admitted to the intensive care units (ICUs) of the Beth Israel Deaconess Medical Center, Boston, USA. The **external-validation cohort** was drawn from the eICU Collaborative Research Database (eICU-CRD) (17), a multi-center database of ICU admissions across the United States, providing a demographically and geographically distinct population for validation. Both databases are publicly available, de-identified, and were accessed under the credentialed-user agreements of the PhysioNet platform (18); their use does not constitute human-subjects research requiring additional ethical approval.

**Institutional validation cohort**. A single-center cohort of critically ill adults with liver disease was assembled from the hospital information system (electronic health records) of Taizhou People's Hospital, a tertiary referral center in Jiangsu Province, China, comprising consecutive ICU admissions between January 2019 and December 2025. Eligibility, the one-record-per-patient rule, the exposure and comparator definitions, lymphocyte truncation and the analytic pipeline were identical to those applied to the public cohorts apart from the laboratory ascertainment window, which is specified in Section 2.3; illness severity was quantified by the Acute Physiology and Chronic Health Evaluation II (APACHE II) score (19), and the harmonized endpoint was in-hospital all-cause mortality, post-discharge vital status not being recorded in the institutional system (as in eICU-CRD). The study was approved by the Taizhou People's Hospital Medical Ethics Committee (approval LSKY 2026-112-01) with a waiver of informed consent given the retrospective design. This approval covered the retrospective analysis of pre-existing, routinely collected electronic health-record data spanning January 2019 to December 2025; all data extraction and analysis were carried out after approval was granted, and no prospective recruitment or post-discharge follow-up was undertaken.

### 2.2 Study population

Eligible patients were adults (aged ≥18 years) with liver disease—defined as cirrhosis or acute/acute-on-chronic liver failure—admitted to an ICU. Liver disease was ascertained from International Classification of Diseases (ICD-9/ICD-10) diagnosis codes in MIMIC-IV and from the structured diagnosis strings in eICU-CRD. For patients with more than one qualifying admission, only the first ICU stay (MIMIC-IV) or first unit stay (eICU-CRD) was retained, so that each cohort contained one record per patient.

**Figure 1:**
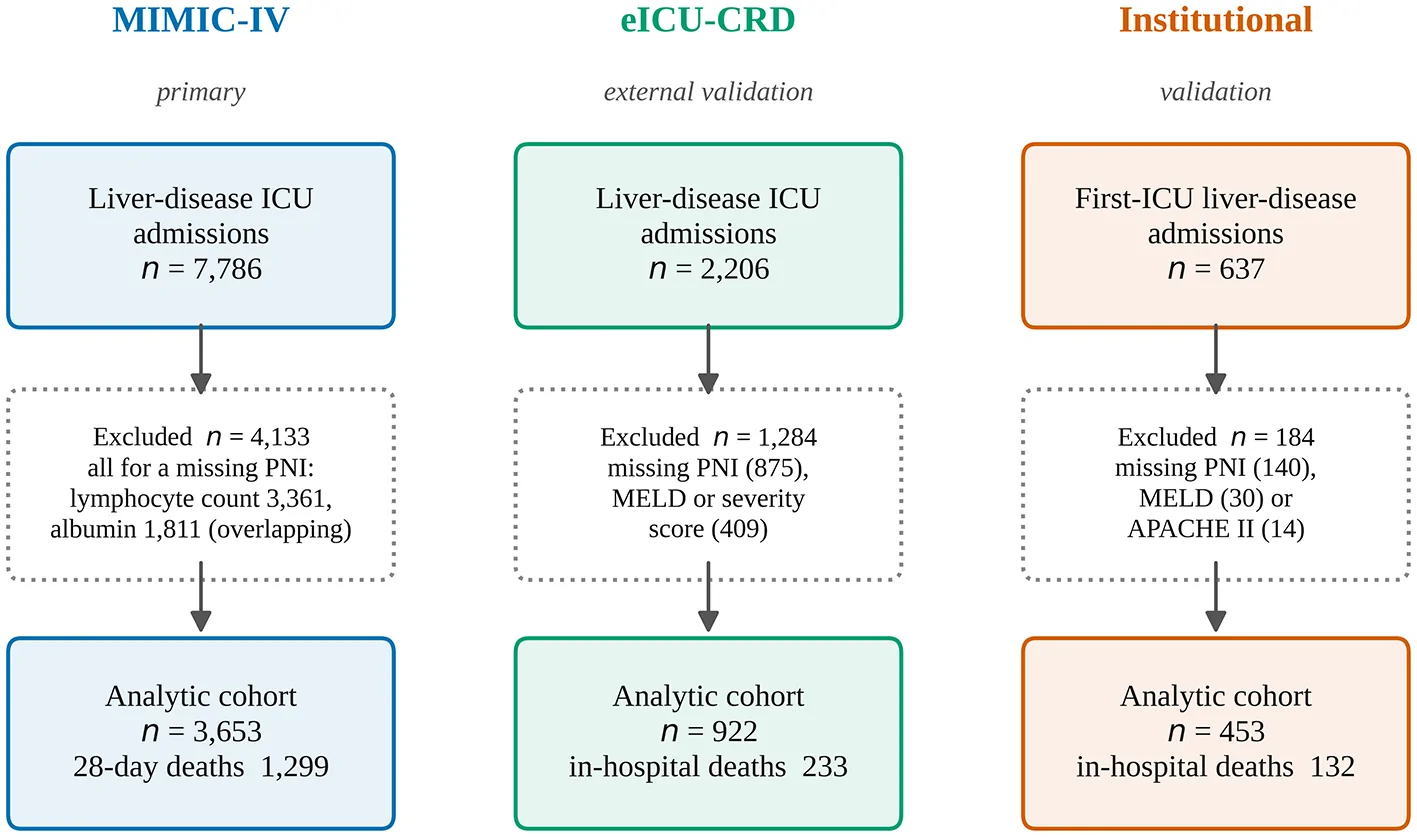
Derivation of the analytic cohorts. Patients with liver disease were identified in MIMIC-IV (*n* = 7,786) and eICU-CRD (*n* = 2,206); after restriction to those with a non-missing Prognostic Nutritional Index, MELD score and severity score, the primary-analysis populations comprised 3,653 patients (MIMIC-IV) and 922 patients (eICU-CRD). The numbers excluded for a missing marker are itemized for each cohort. In MIMIC-IV every exclusion followed from a missing PNI (the lymphocyte differential was absent in 3,361 patients and albumin in 1,811, categories that overlap); in eICU-CRD 875 exclusions followed from a missing PNI and a further 409 from a missing MELD component or severity score. In the single-center institutional cohort (Taizhou People's Hospital), 637 first-ICU liver-disease admissions yielded 453 patients after the same restriction (140 excluded for a missing PNI component, 30 for a missing MELD component and 14 for a missing APACHE II score). PNI, Prognostic Nutritional Index; MELD, Model for End-Stage Liver Disease; ICU, intensive care unit.

Applying these criteria yielded a liver-disease source population of **7,786 patients** in MIMIC-IV (cirrhosis, *n* = 5,552; liver failure, *n* = 3,876; the two categories overlap) and **2,206 patients** in eICU-CRD (cirrhosis, *n* = 1,656; liver failure, *n* = 810). The primary analyses used the complete-case population for the principal exposure–comparator set—that is, patients with non-missing PNI, MELD and a severity score—comprising **3,653 patients** in MIMIC-IV and **922 patients** in eICU-CRD; the derivation of these analytic samples from the source populations is summarized in Figure 1. To avoid mixing analytic populations within the main text, all main-text analyses fixed one population per cohort (MIMIC-IV, *n* = 3,653; eICU-CRD, *n* = 922); the wider marker-available subsets were reserved for sensitivity analyses reported in the Supplementary materials. In the institutional cohort the same procedure yielded 637 first-ICU liver-disease admissions, of which **453 patients** had a non-missing PNI, MELD score and APACHE II score and formed the institutional primary-analysis population (Figure 1).

### 2.3 Exposure, comparators and covariates

The **exposure** of interest was the Prognostic Nutritional Index, calculated with the original Onodera formula (6):

PNI = 10 × serum albumin (g/dL) + 0.005 × total lymphocyte count (cells/μL).

Both inputs carry a recognized nutritional interpretation—serum albumin as a marker of visceral protein status and the lymphocyte count as a marker of immunonutritional reserve—yet both are also strongly influenced by systemic inflammation and acute physiological stress, and current nutritional-assessment frameworks (for example, the Global Leadership Initiative on Malnutrition) (4) no longer endorse albumin as a stand-alone marker of nutritional status for this reason.

The principal **comparator** was MELD (11), the international reference for prognostication and organ allocation in liver disease, computed as

MELD = 9.57 × ln(creatinine, mg/dL) + 3.78 × ln(total bilirubin, mg/dL) + 11.20 × ln(INR) + 6.43,

with each laboratory value floored at 1.0 before logarithmic transformation and serum creatinine capped at 4.0 mg/dL, per the standard convention; for patients receiving renal-replacement therapy the creatinine was likewise set to the 4.0 mg/dL ceiling. The original MELD was used as the primary comparator in preference to MELD 3.0 (12), which incorporates serum albumin and would directly confound a comparison whose explicit purpose is to isolate the value of an albumin-based index; the incremental value of the PNI over MELD 3.0 is reported below as a sensitivity analysis (Supplementary Table S10). MELD-Na (which adds serum sodium, not albumin) does not share this problem but was not adopted as the primary comparator, so that the reference remained the most parsimonious internationally standard formulation. Two contemporary MELD variants were examined as sensitivity comparators. MELD-Na was computed by the standard correction (20), MELD-Na = MELD + 1.32 × (137 – Na) – 0.033 × MELD × (137 – Na), applied when MELD exceeded 11, with sodium bounded to 125–137 mmol/L; MELD 3.0 was computed from the published coefficients, including the sex term and the albumin-by-creatinine interaction, with creatinine bounded to 1.0–3.0 mg/dL, bilirubin and the international normalized ratio floored at 1.0, albumin bounded to 1.5–3.5 g/dL and sodium bounded to 125–137 mmol/L. Both were truncated to the range 6–40. In MIMIC-IV both were derived from the same laboratory inputs as the MELD reported above, so that the three scores are strictly comparable; in eICU-CRD the components required for MELD 3.0 were re-extracted for this analysis, and MELD recomputed from them agreed closely with the score used in the main analysis (r = 0.96). In the institutional cohort, C-reactive protein and procalcitonin were additionally retrieved as the first available value within 72 h of ICU admission; their availability was also quantified in the two public cohorts. Two additional albumin-related comparators were predefined: serum **albumin alone** (to test whether adding the lymphocyte term to albumin improves prognostication) and the **blood urea nitrogen–to–albumin ratio (BAR)**. Red-cell distribution width (**RDW**), a non–albumin-based, routinely available hematological marker, was predefined as an exploratory comparator.

For each patient, PNI, albumin, RDW and BAR were derived from the first available laboratory value obtained within 72 h of ICU admission; in MIMIC-IV and eICU-CRD, values recorded up to 6 h before ICU admission were also eligible, so as to capture emergency-department and pre-ICU sampling, whereas the institutional cohort used measurements obtained after ICU admission only, because the hospital information system does not carry a timestamp for samples drawn before the patient reaches the unit. In MIMIC-IV, MELD was obtained from the corresponding derived concept of the MIMIC Code Repository, which applies the formula above to the most abnormal value of each component recorded on the first ICU day; that concept substitutes the conventional lower bound of 1.0 when a component is not recorded, which applied to 137 of the 3,653 analyzed patients (3.8%), and restricting the analysis to the 3,569 patients with all three components recorded within the ascertainment window gave a MELD area under the curve of 0.697 against 0.699 overall (Supplementary Table S14). In eICU-CRD and the institutional cohort, where no such derived concept exists, MELD was computed directly from the formula above using the first value of each component in the same window as the PNI, so that in those two cohorts the two scores are ascertained by an identical rule. **Illness severity** was summarized by the Simplified Acute Physiology Score II (SAPS-II) (21) in MIMIC-IV and, because SAPS-II is not available in eICU-CRD, by the Acute Physiology and Chronic Health Evaluation IV (APACHE IV) (22) score in eICU-CRD (available in approximately 86% of the full eICU-CRD extraction; the primary analysis, which required a severity score, was restricted to patients with a recorded APACHE IV); in the institutional cohort, severity was quantified by the APACHE II score. In MIMIC-IV, the following comorbidities were additionally captured for descriptive and sensitivity purposes: diabetes mellitus, chronic renal disease, malignancy and the Charlson Comorbidity Index (23). Acute conditions comprised sepsis [Sepsis-3 criteria (24)], acute kidney injury graded by the KDIGO definition (25), and the Oxford Acute Severity of Illness Score [OASIS, reported descriptively (26)].

Because the two public cohorts report laboratory results in different units, all values were harmonized to a common scale before any index was computed: serum albumin to g/dL (dividing g/L by 10), absolute lymphocyte count to cells/μL (multiplying 10^9^/L by 1,000), total bilirubin to mg/dL (dividing μmol/L by 17.1), and creatinine to mg/dL (dividing μmol/L by 88.4). The full cross-cohort variable-definition and unit-conversion specification, including the institutional source fields, is provided in Supplementary Table S1. Cross-cohort consistency of the harmonized inputs was confirmed before analysis (Supplementary materials). Implausible total lymphocyte counts were truncated to the physiological range of 100–10,000 cells/μL before computing PNI (affecting approximately 1–3% of values, for example concurrent hematological malignancy), so that a few extreme counts did not distort variance-based statistics; rank-based discrimination measures (the AUC analyses) were unaffected by this step.

### 2.4 Outcomes

The **primary outcome** was all-cause mortality within 28 days of ICU admission in the primary cohort; in MIMIC-IV, 28-day vital status was ascertained from the linked out-of-hospital date-of-death record, so that deaths occurring after hospital discharge were also captured. Because eICU-CRD does not record vital status after hospital discharge, the harmonized endpoint for **external validation** was in-hospital all-cause mortality; the 28-day endpoint was therefore retained for the primary cohort and the validation cohort was assessed at the in-hospital level, the two cohorts being compared on the dimension on which they are jointly measurable. **Secondary outcomes** in the primary cohort were in-hospital mortality and 90-day mortality. In the **institutional validation cohort**, as in eICU-CRD, only in-hospital all-cause mortality was available and analyzed; post-discharge follow-up was not feasible in this retrospective single-center extraction.

### 2.5 Statistical analysis

Continuous variables are summarized as median (interquartile range, IQR) and categorical variables as count (percentage). The analysis addressed three questions in sequence: how well each marker discriminates death; whether PNI adds prognostic information beyond MELD; and why PNI behaves as it does.

**Discrimination** was quantified by the area under the receiver-operating-characteristic curve (AUC) for each marker, with 95% confidence intervals (CIs) obtained from the DeLong variance estimator (27). Markers were oriented so that a higher predicted value corresponded to a higher risk of death; for protective markers (PNI, albumin) the curve was constructed on the negated value. Pairwise differences in AUC—specifically MELD versus PNI, and PNI versus albumin alone—were tested with the DeLong test for two correlated ROC curves on the subset of patients with both markers available.

**Associations** with mortality were estimated by logistic regression and expressed as odds ratios (ORs) per one–standard-deviation (SD) increment of each marker, both unadjusted and adjusted for MELD, age and the cohort-specific severity score (SAPS-II in MIMIC-IV, APACHE IV in eICU-CRD, APACHE II in the institutional cohort). The **incremental value** of PNI was assessed by adding PNI to a model already containing MELD and examining the change in the C-statistic (tested with the DeLong method) and the integrated discrimination improvement (IDI) (28), together with the adjusted PNI coefficient. As a predefined sensitivity analysis, PNI was also modeled dichotomously at the classical threshold of < 45.

To probe the **mechanism** underlying PNI's behavior, the discrimination of PNI for the primary outcome was estimated separately within strata of liver-disease severity defined *a priori* by MELD (< 15, 15 to < 25, and ≥ 25). Modification of PNI's association with mortality by severity was, in addition, tested formally with a PNI × MELD interaction term.

Analyses were performed in R (version 4.5.2; R Foundation for Statistical Computing, Vienna, Austria). Logistic-regression models were fitted by maximum likelihood with the glm function (binomial family). Areas under the receiver-operating-characteristic curve and their 95% confidence intervals, and the comparison of two correlated curves, were obtained with the pROC package (version 1.18 or later); the DeLong test for two correlated areas under the curve follows the algorithm of Sun and Xu (29). Analysis code is openly available in a public repository (Zenodo; https://doi.org/10.5281/zenodo.20894993). The principal analyses were complete-case; the robustness of the findings to this choice was examined in the Supplementary materials by repeating the marker analyses in the wider marker-available subsets (Supplementary Table S2) and by comparing complete and incomplete cases (Supplementary Table S3). To gauge sensitivity to unmeasured confounding, E-values were computed for the adjusted associations (30) (Supplementary Table S5). The identical analytic pipeline—discrimination, per-SD associations, incremental value over MELD, and severity-stratified analysis—was applied to the institutional cohort using the harmonized definitions in Supplementary Table S1 and is reported alongside the public cohorts. Five analyses were added in this revision; the definitions of MELD-Na and MELD 3.0 are given in Section 2.3 and their results in Supplementary Table S10. First, to address the proportion of patients excluded for missing data, the marker analyses were repeated in the full source populations of both public cohorts after multiple imputation by chained equations, implemented with the mice package (31) (*m* = 10; imputation model containing albumin, lymphocyte count, RDW, urea, MELD, the cohort-specific severity score, age, sex, diagnosis category, the outcome and, in MIMIC-IV, the Charlson Comorbidity Index), with estimates pooled by Rubin's rules. Second, to examine whether the index carries different prognostic information once the acute phase has partly resolved, the PNI was recomputed from the first albumin and lymphocyte values obtained between 72 h and 7 days after ICU admission and compared with the baseline value within the same patients. Third, in the institutional cohort the associations were re-examined with adjustment for log-transformed C-reactive protein and procalcitonin, and correlations between the inflammatory markers and the PNI and its components were quantified by Spearman coefficients. Fourth, because MELD in MIMIC-IV is derived from the most abnormal value of each component on the first ICU day whereas the PNI uses the first value in the ascertainment window, MELD was recomputed from the first value of each component in the same window and its discrimination compared with the primary derivation. All tests were two-sided with a significance threshold of 0.05; given the explicitly comparative and partly exploratory aims, emphasis is placed on effect sizes and confidence intervals rather than on isolated *p*-values, and the RDW findings are reported as exploratory.

## 3 Results

### 3.1 Patients and baseline characteristics

Of the 7,786 patients with liver disease in MIMIC-IV, the 2,206 in eICU-CRD and the 637 first-ICU liver-disease admissions at the institutional center, 3,653, 922 and 453 respectively had a non-missing PNI, MELD score and severity score and constituted the primary-analysis populations (Figure 1; Supplementary Table S4 compares the analyzed and excluded institutional cases). The cohorts all represented advanced critical illness. The median MELD score was 25 (IQR 18–33) in MIMIC-IV and 21 (16–29) in eICU-CRD, and mortality was high: 28-day mortality reached 35.6% (1,299/3,653) in the primary cohort, while in-hospital mortality was 32.0% (1,170/3,653) in MIMIC-IV, 25.3% (233/922) in eICU-CRD and 29.1% (132/453) in the institutional cohort; 28-day mortality exceeded in-hospital mortality in MIMIC-IV because the linked date-of-death record captured deaths after discharge. Cirrhosis was present in 66.4% and 73.6% of patients and acute or acute-on-chronic liver failure in 57.0% and 40.7%, respectively (the two categories overlap and are not mutually exclusive). Consistent with severe hepatic dysfunction, both the median serum albumin [2.9 g/dL (2.5–3.4) and 2.5 g/dL (2.0–2.9)] and the median PNI [34.6 (29.3–40.0) and 30.1 (25.5–35.3)] were low and lay well below the conventional PNI “malnutrition” threshold of 45, which classified 89.5%, 95.2% and 96.9% of the primary, external-validation and institutional cohorts as abnormal.

The institutional cohort, while showing the same advanced critical illness, differed in case-mix from the two North American databases: its liver disease was most commonly hepatitis-B-related (hepatitis B accounting for 48.1% of cirrhosis etiology, against an alcohol-predominant profile in MIMIC-IV and eICU-CRD; Supplementary Table S8), and its patients had the lowest median serum albumin [2.4 g/dL (2.0–2.8)] and PNI [28.3 (23.2–33.4)] of the three cohorts, consistent with a more profoundly hypoalbuminaemic population; its median MELD (22) and in-hospital mortality (29.1%) lay between those of the two public cohorts. Full baseline characteristics for all three cohorts are shown in Table 1.

**Table 1:** Baseline characteristics of the analytic cohorts.

Characteristic	MIMIC-IV (*n* = 3,653)	eICU-CRD (*n* = 922)	Institutional (*n* = 453)
Age, years	60 (51–69)	56 (49–64)	55 (46–65)
Male sex	2,258 (61.8)	569 (61.7)	296 (65.3)
Cirrhosis	2,425 (66.4)	679 (73.6)	345 (76.2)
Acute/acute-on-chronic liver failure	2,084 (57.0)	375 (40.7)	193 (42.6)
Diabetes mellitus	1,130 (30.9)	—	100 (22.1)
Chronic renal disease	850 (23.3)	—	81 (17.9)
Malignancy	556 (15.2)	—	85 (18.8)
Sepsis (Sepsis-3)	2,082 (57.0)	—	217 (47.9)
Acute kidney injury (any stage)	3,016 (82.6)	—	332 (73.3)
MELD score	25 (18–33)	21 (16–29)	22 (16–30)
SAPS-II/APACHE IV/APACHE II	43 (32–55)	75 (58–98)	18 (13–24)
OASIS	34 (28–42)	—	—
Charlson Comorbidity Index	5 (4–8)	—	5 (3–7)
Serum albumin, g/dL	2.9 (2.5–3.4)	2.5 (2.0–2.9)	2.4 (2.0–2.8)
Lymphocytes, cells/μL	880 (520–1,440)	878 (523–1,420)	810 (470–1,340)
Prognostic Nutritional Index	34.6 (29.3–40.0)	30.1 (25.5–35.3)	28.3 (23.2–33.4)
RDW, %	16.3 (14.7–18.7)	16.9 (15.3–19.0)	16.1 (14.5–18.2)
BAR	10.0 (5.8–16.7)	11.4 (6.3–18.6)	9.8 (6.2–16.4)
Blood urea nitrogen, mg/dL	28 (17–48)	26 (16–46)	24 (14–43)
ICU stay, days	3.0 (1.5–6.6)	—	4.1 (2.0–8.3)
Hospital stay, days	11 (5–20)	—	14 (7–24)
In-hospital death	1,170 (32.0)	233 (25.3)	132 (29.1)
28-day death	1,299 (35.6)	—	—
90-day death	1,638 (44.8)	—	—

Data are median (interquartile range) for continuous variables and n (%) for categorical variables. Illness severity was quantified by SAPS-II in MIMIC-IV, APACHE IV in eICU-CRD and APACHE II in the institutional cohort; these are different scales and are not directly comparable across cohorts. The cirrhosis and liver-failure categories overlap and are not mutually exclusive. “—” denotes a variable not recorded in that database (post-discharge mortality is unavailable in eICU-CRD and the institutional cohort). PNI, Prognostic Nutritional Index; MELD, Model for End-Stage Liver Disease; SAPS-II, Simplified Acute Physiology Score II; APACHE, Acute Physiology and Chronic Health Evaluation; OASIS, Oxford Acute Severity of Illness Score; RDW, red-cell distribution width; BAR, blood urea nitrogen–to–albumin ratio; ICU, intensive care unit.

### 3.2 Discrimination of PNI and comparator markers

**Figure 2:**
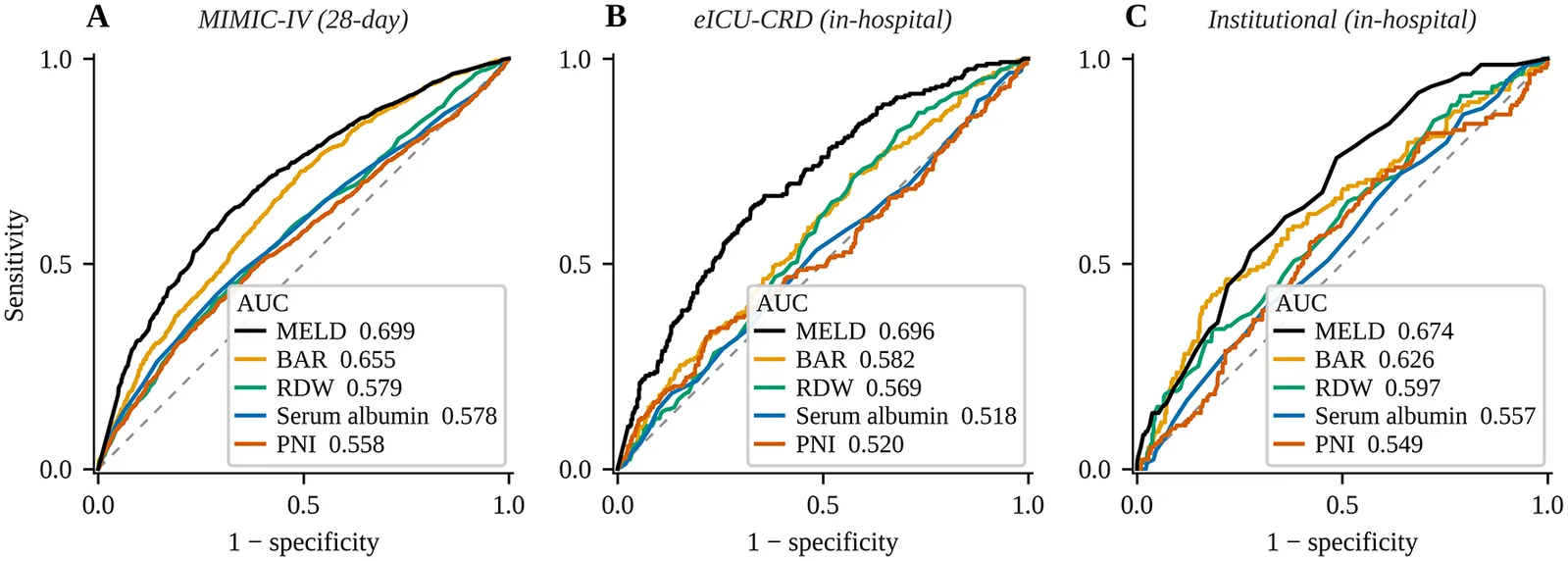
Empirical receiver-operating-characteristic curves for the discrimination of mortality by PNI, serum albumin, RDW, BAR and MELD, computed from patient-level data in all three cohorts. **(A)** 28-day mortality, MIMIC-IV primary cohort (*n* = 3,653). **(B)** In-hospital mortality, eICU-CRD external-validation cohort (*n* = 922). **(C)** In-hospital mortality, institutional validation cohort (*n* = 453). Protective markers were oriented so that higher plotted values indicate higher risk. The AUC for each marker is shown in the panel legend, with the full set of AUCs and pairwise DeLong comparisons given in Table 2. Abbreviations as in Table 1.

MELD discriminated mortality moderately well and consistently across cohorts, with an AUC of **0.699** (95% CI 0.681–0.717) for 28-day mortality in MIMIC-IV and **0.696** (0.658–0.734) for in-hospital mortality in eICU-CRD. By contrast, PNI discriminated little better than chance: the AUC was **0.558** (0.538–0.578) in the primary cohort and **0.520** (0.475–0.565) in the validation cohort. Serum albumin alone performed essentially identically to PNI [0.578 (0.558–0.598) and 0.518 (0.474–0.561)], as did the exploratory hematological marker RDW [0.579 (0.560–0.599) and 0.569 (0.528–0.611)]. BAR was intermediate [0.655 (0.637–0.673) and 0.582 (0.540–0.624)] but remained inferior to MELD in both cohorts. The institutional cohort reproduced this ordering: PNI 0.549 (0.498–0.600), albumin 0.557 (0.506–0.608), RDW 0.597 (0.547–0.647) and BAR 0.626 (0.577–0.675), with MELD [0.674 (0.625–0.723)] again the only marker reaching moderate discrimination. The receiver-operating-characteristic curves are shown in Figure 2 and the AUCs in Table 2.

**Table 2:** Discrimination of mortality by each marker across the three cohorts.

Marker	MIMIC-IV—AUC (95% CI)	eICU-CRD—AUC (95% CI)	Institutional—AUC (95% CI)
PNI	0.558 (0.538–0.578)	0.520 (0.475–0.565)	0.549 (0.498–0.600)
Serum albumin	0.578 (0.558–0.598)	0.518 (0.474–0.561)	0.557 (0.506–0.608)
RDW	0.579 (0.560–0.599)	0.569 (0.528–0.611)	0.597 (0.547–0.647)
BAR	0.655 (0.637–0.673)	0.582 (0.540–0.624)	0.626 (0.577–0.675)
MELD	0.699 (0.681–0.717)	0.696 (0.658–0.734)	0.674 (0.625–0.723)
**MELD** **+** **PNI (combined)**	0.702 (0.685–0.720)	0.695 (0.657–0.734)	0.679 (0.630–0.728)

Area under the receiver-operating-characteristic curve (AUC) with 95% confidence interval (DeLong estimator). Outcomes are 28-day mortality (MIMIC-IV) and in-hospital mortality (eICU-CRD and the institutional cohort). Protective markers (PNI, albumin) were oriented so that lower values denote higher risk. The final row is a two-variable logistic model; adding PNI to MELD did not improve discrimination (change in C-statistic +0.003, DeLong p = 0.06, integrated discrimination improvement [IDI] = 0.0034 in MIMIC-IV; −0.001, p = 0.71, IDI = 0.0004 in eICU-CRD; +0.005, p = 0.48, IDI = 0.0021 in the institutional cohort). Marker-specific denominators reduced by missing data: RDW n = 3,648 (MIMIC-IV), 879 (eICU-CRD) and 446 (institutional); BAR n = 3,651 (MIMIC-IV) and all patients in the other two cohorts; all remaining cells use the full populations. Pairwise DeLong comparisons—MELD vs PNI: ΔAUC = 0.141 (p = 1.7 × 10^−24^), 0.176 (p = 7.2 × 10^−9^) and 0.125 (p = 5.1 × 10^−5^); PNI vs albumin alone: ΔAUC = −0.020 (p = 8.8 × 10^−4^), +0.002 (p = 0.88) and −0.008 (p = 0.64), in MIMIC-IV, eICU-CRD and the institutional cohort respectively. Abbreviations as in Table 1.

On formal comparison, MELD discriminated significantly better than PNI in both cohorts—an AUC difference of **0.141** (DeLong *p* = 1.7 × 10^−24^) in MIMIC-IV and **0.176** (*p* = 7.2 × 10^−9^) in eICU-CRD. Critically, adding the lymphocyte term to albumin—the sole difference between PNI and albumin alone—did not improve discrimination: PNI was marginally **worse** than albumin in the primary cohort (ΔAUC −0.020, *p* = 8.8 × 10^−4^) and statistically indistinguishable from it in the validation cohort (ΔAUC +0.002, *p* = 0.88). In the institutional cohort the same two results held—MELD again discriminated far better than PNI (ΔAUC 0.125, *p* = 5.1 × 10^−5^) and PNI was again indistinguishable from albumin alone (ΔAUC −0.008, *p* = 0.64). PNI therefore captured no prognostic signal beyond that already present in its albumin component. Because the two cohorts were assessed on different endpoints (28-day vs in-hospital mortality), the analysis was repeated in MIMIC-IV using in-hospital mortality to match eICU-CRD exactly; discrimination was virtually identical (PNI AUC 0.556, MELD AUC 0.697), indicating that the cross-cohort contrast is not an artifact of the endpoint definition.

### 3.3 PNI does not outperform albumin or add to MELD

**Figure 3:**
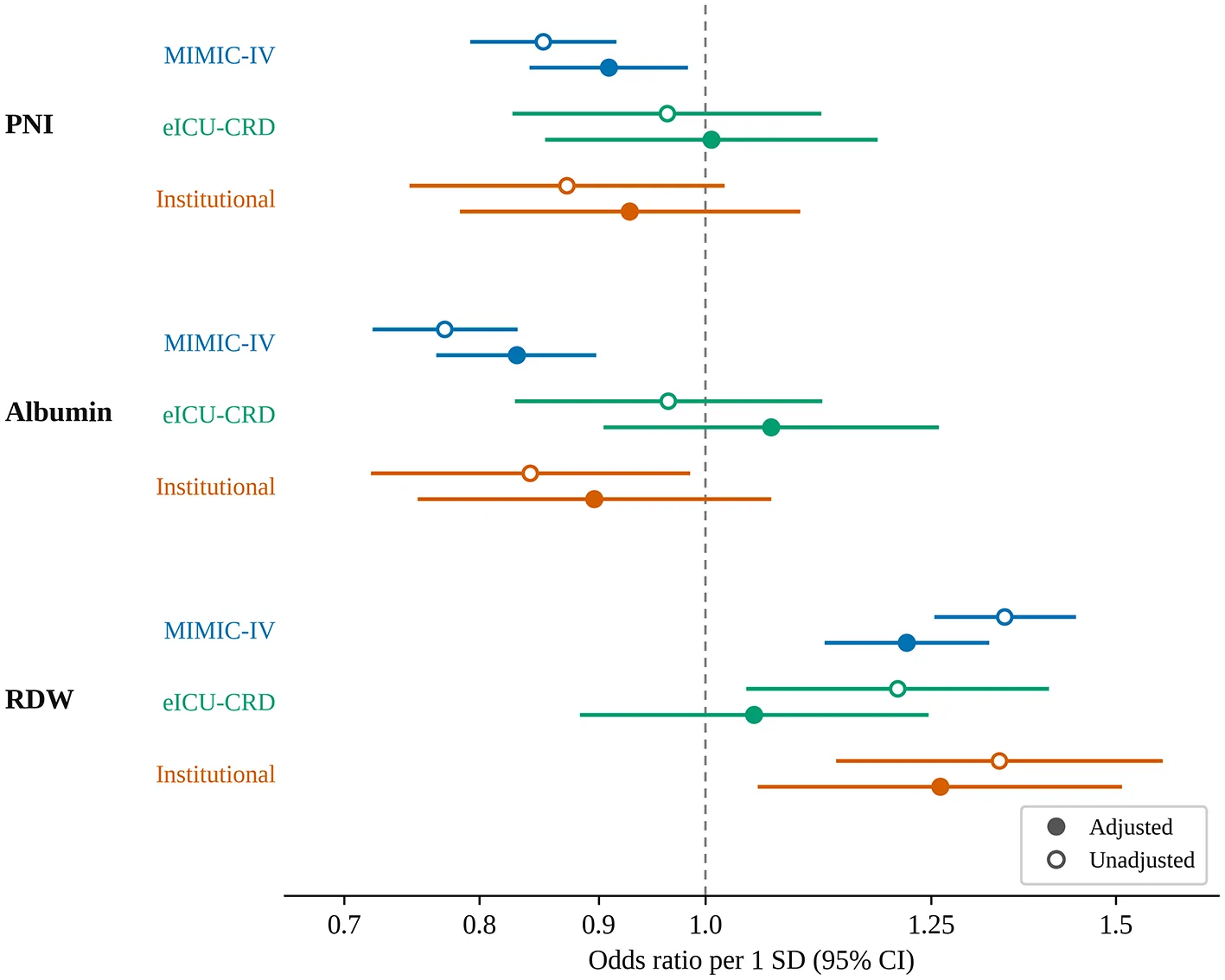
Forest plot of odds ratios for mortality per one–standard-deviation increment of PNI, serum albumin and RDW, before and after adjustment for MELD, age and the cohort-specific severity score, in the MIMIC-IV (28-day mortality), eICU-CRD (in-hospital mortality) and institutional (in-hospital mortality) cohorts. Circles denote point estimates (filled, adjusted; open, unadjusted) and horizontal lines the 95% confidence intervals; the vertical reference line marks an odds ratio of 1. Estimates are listed in Table 3. Abbreviations as in Table 1.

Per one–standard-deviation (SD) increment, PNI showed at most a weak association with mortality. In MIMIC-IV the unadjusted per-SD odds ratio (OR) for 28-day mortality was 0.852 (0.794–0.914, *p* < 0.001) and remained weakly but significantly associated after adjustment for MELD, age and SAPS-II (adjusted OR 0.909, 0.842–0.981, *p* = 0.014). In eICU-CRD, however, PNI was unassociated with in-hospital mortality both before (OR 0.963, 0.828–1.119, *p* = 0.62) and after adjustment (1.006, 0.855–1.183, *p* = 0.95), so even this weak association did not replicate externally; because the unadjusted estimate was already null, this absence of association cannot be attributed to the serum albumin that the APACHE IV score used for adjustment in eICU-CRD partly incorporates. Serum albumin behaved similarly—an independent inverse association in MIMIC-IV (adjusted OR 0.830, 0.768–0.896, *p* < 0.001) but none in eICU-CRD (1.067, 0.906–1.257, *p* = 0.44)—whereas RDW retained an independent association in the primary cohort (adjusted OR 1.220, 1.127–1.321, *p* < 0.001; *n* = 3,648). The institutional cohort followed the external-validation pattern: after adjustment PNI was non-significant (OR 0.928, 0.786–1.096, *p* = 0.38), as was albumin (0.896, 0.754–1.065, *p* = 0.21), whereas RDW retained an independent association (1.261, 1.055–1.506, *p* = 0.011). The per-SD estimates are shown in Table 3 and Figure 3.

**Table 3:** Association of each marker with mortality per one–standard-deviation increment, unadjusted and adjusted.

Marker	Cohort	Unadjusted OR (95% CI)	Adjusted OR (95% CI)
PNI	MIMIC-IV	0.852 (0.794–0.914), *p* < 0.001	0.909 (0.842–0.981), *p* = 0.014
	eICU-CRD	0.963 (0.828–1.119), *p* = 0.62	1.006 (0.855–1.183), *p* = 0.95
	Institutional	0.872 (0.748–1.017), *p* = 0.08	0.928 (0.786–1.096), *p* = 0.38
Serum albumin	MIMIC-IV	0.773 (0.721–0.829), *p* < 0.001	0.830 (0.768–0.896), *p* < 0.001
	eICU-CRD	0.964 (0.830–1.120), *p* = 0.63	1.067 (0.906–1.257), *p* = 0.44
	Institutional	0.841 (0.720–0.983), *p* = 0.03	0.896 (0.754–1.065), *p* = 0.21
RDW	MIMIC-IV	1.344 (1.256–1.439), *p* < 0.001	1.220 (1.127–1.321), *p* < 0.001
	eICU-CRD	1.209 (1.043–1.401), *p* = 0.012	1.049 (0.885–1.244), *p* = 0.58
	Institutional	1.337 (1.140–1.568), *p* < 0.001	1.261 (1.055–1.506), *p* = 0.011

Odds ratios per one–standard-deviation increment from logistic regression. Adjusted models include MELD, age and the cohort-specific severity score (SAPS-II in MIMIC-IV, APACHE IV in eICU-CRD, APACHE II in the institutional cohort). Outcomes are 28-day mortality (MIMIC-IV) and in-hospital mortality (eICU-CRD and the institutional cohort). RDW analyses: MIMIC-IV n = 3,648 (1,298 events), eICU-CRD n = 879 (220 events), institutional n = 446 (130 events); PNI and albumin analyses use the full populations (MIMIC-IV n = 3,653; eICU-CRD n = 922; institutional n = 453). One standard deviation corresponded to 8.7 (PNI), 0.68 g/dL (albumin) and 9.5 (MELD) in MIMIC-IV, 8.5, 0.72 and 8.7 in eICU-CRD, and 7.5, 0.59 and 9.7 in the institutional cohort; PNI was computed from lymphocyte counts truncated to 100–10,000 cells/μL (see Methods), so the PNI SDs are based on the truncated scale. The PNI standard deviations are similar across cohorts, and repeating the per-SD analysis with a common standard deviation left the estimates essentially unchanged (for example, the eICU-CRD adjusted PNI OR was 1.006 on either scale). SD, standard deviation; OR, odds ratio; other abbreviations as in Table 1.

Crucially, this weak association did not translate into clinical value. Adding PNI to a logistic model already containing MELD changed discrimination negligibly: the C-statistic moved from 0.699 to 0.702 in MIMIC-IV [ΔC +0.003, 95% CI −0.001 to +0.007, *p* = 0.06; integrated discrimination improvement (IDI) = 0.0034] and from 0.696 to 0.695 in eICU-CRD (*p* = 0.71) and from 0.674 to 0.679 in the institutional cohort (ΔC +0.005, *p* = 0.48; IDI = 0.0021), and even the upper confidence bound of the increment (about +0.007 AUC units) is clinically negligible (Table 2, final row). The marginally negative change in eICU-CRD is not paradoxical: the apparent C-statistic of a fitted model need not increase when an uninformative term is added, since the AUC is not the quantity the likelihood optimizes. Because PNI and MELD were almost completely uncorrelated (Pearson *r* = −0.055, −0.003 and −0.038), this lack of incremental value reflects an absence of clinically useful prognostic information rather than redundancy with MELD.

The same held against the two contemporary MELD variants. Serum sodium was available for all 3,653 MIMIC-IV and all 922 eICU-CRD analytic patients, so MELD-Na could be computed for all of them and MELD 3.0 for 3,516 (96.2%) and 922 respectively, the 137 exceptions lacking a recorded bilirubin or international normalized ratio on the first ICU day (a different denominator from the 3,569 patients with all three components available within the wider ascertainment window, Supplementary Table S14). Both variants discriminated comparably to the original MELD in MIMIC-IV (0.694 and 0.696 versus 0.699) and, in eICU-CRD, MELD-Na comparably and MELD 3.0 somewhat lower (0.689 and 0.663 versus 0.696) and both exceeded the PNI by a wide margin (ΔAUC 0.136–0.169, all *p* < 10^−5^). Adding the PNI changed the C-statistic by +0.0035 (*p* = 0.06) over MELD, by +0.0031 (*p* = 0.09) over MELD-Na and by +0.0017 (*p* = 0.29) over MELD 3.0 in MIMIC-IV, and by −0.00005 (*p* = 0.96) and +0.0004 (*p* = 0.50) in eICU-CRD (Supplementary Table S10). In MIMIC-IV the increment was smallest over MELD 3.0, the only variant that already contains serum albumin; in eICU-CRD all three increments were indistinguishable from zero (−0.0006 to +0.0004) and their ordering is not interpretable.

In MIMIC-IV, MELD and the PNI are not ascertained by an identical rule: the MELD components are the most abnormal values of the first ICU day, whereas the PNI uses the first value in the ascertainment window. Recomputing MELD from the first value of each component in that same window lowered its discrimination only slightly (0.685, 95% CI 0.667–0.704, against 0.697 for the primary derivation in the same 3,569 patients) and left its advantage over the PNI essentially intact (ΔAUC 0.126, *p* = 4.9 × 10^−19^). The ascertainment rule therefore accounts for about 0.012 AUC units, an order of magnitude less than the difference between MELD and the PNI (Supplementary Table S14).

Dichotomising PNI at the classical threshold of < 45 did not recover prognostic value: the abnormal category carried an OR for death of 0.929 (0.746–1.157, *p* = 0.51) in MIMIC-IV, 0.712 (0.371–1.368, *p* = 0.31) in eICU-CRD and 1.03 (0.32–3.34, *p* = 0.96) in the institutional cohort. None of the estimates was significant, and all are unstable because a low PNI was nearly universal in these severe populations (89.5%, 95.2% and 96.9% of patients fell below the threshold), leaving only a small normal-PNI reference group; the dichotomy is therefore uninformative in this setting. The secondary outcomes were consistent with the primary: for 90-day mortality in MIMIC-IV the adjusted per-SD OR for PNI remained weak and, if anything, marginally stronger than at 28 days (0.883, 0.820–0.951, *p* < 0.001), as were albumin (0.823, 0.764–0.887, *p* < 0.001) and RDW (1.291, 1.195–1.396, *p* < 0.001). Thus, although PNI was weakly associated with mortality in the primary cohort, the association did not replicate in external validation, PNI did not outperform its own albumin component, and it added no clinically meaningful discrimination beyond MELD for the primary or in-hospital outcomes.

### 3.4 Factors associated with the weak discrimination of the PNI

**Figure 4:**
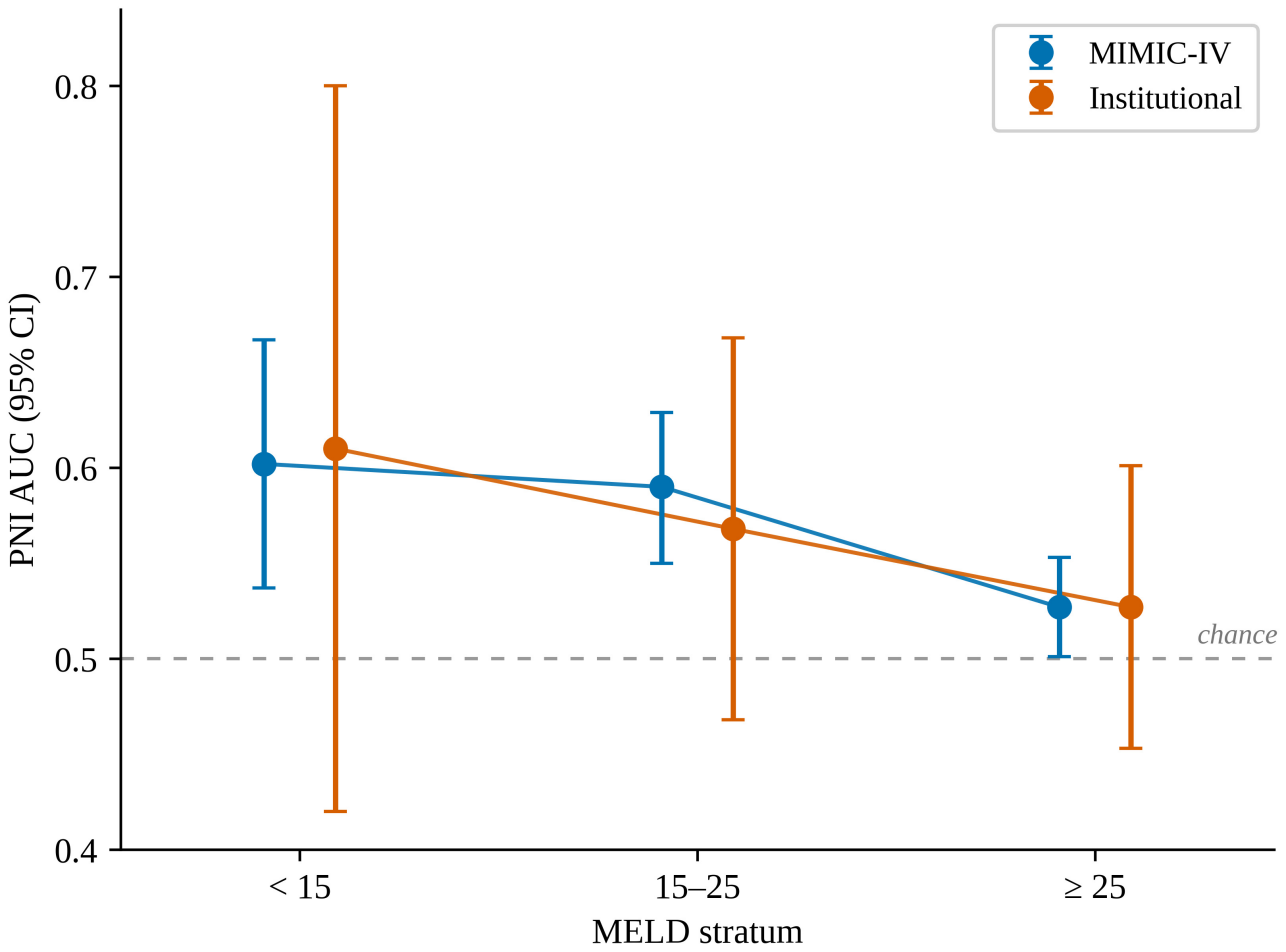
Discrimination (AUC, with 95% confidence interval) of PNI for mortality within prespecified MELD strata, shown for the MIMIC-IV cohort (28-day mortality; blue) and the institutional cohort (in-hospital mortality; orange). The dashed horizontal line indicates non-informative discrimination (AUC = 0.5). In MIMIC-IV, PNI discrimination falls from 0.602 in mild disease (MELD < 15) to 0.527 in severe disease (MELD ≥ 25); the institutional cohort shows a similar pattern (0.610 to 0.527) with much wider intervals reflecting its smaller size. Confidence intervals overlap across strata in both cohorts, indicating an imprecise gradient. Stratum sizes and event counts are given in Table 4 (MIMIC-IV) and Supplementary Table S7 (institutional). Abbreviations as in Table 1.

Two observations help to characterize the weak discrimination of the PNI in this setting. First, PNI was almost orthogonal to MELD (Pearson r ≈ −0.06), so it carries essentially no information about the acute organ failure that drives short-term mortality in decompensated liver disease—the dimension MELD captures. This near-orthogonality is consistent with the constructs the two indices are built from: MELD is built from bilirubin, creatinine and the international normalized ratio, which index acute hepatic and renal failure, whereas the albumin and lymphocyte components of PNI have been reported to track chronic synthetic reserve and systemic inflammation (9, 10); the narrow range of both components in this uniformly severe population further constrains any shared variation. Consistent with this, the association of PNI with 28-day mortality was modified by severity (PNI × MELD interaction *p* = 0.03), and discrimination was weakest in the most severe stratum (MELD ≥ 25: AUC 0.527, 95% CI 0.501–0.553), where most deaths occurred—a point estimate close to chance, with a confidence interval whose lower bound only marginally exceeds 0.5 (Table 4, Figure 4). Discrimination in the two lower-severity strata was only modestly higher and imprecise (MELD < 15: 0.602, 0.537–0.667; MELD 15 to < 25: 0.590, 0.550–0.629), and the overall AUC (0.558) lies below these point estimates, consistent with pooling across strata of differing baseline risk—which PNI does not track—diluting rather than adding to the within-stratum signal. The same severity gradient was reproduced in the institutional cohort (PNI AUC 0.610, 0.568 and 0.527 across the three MELD strata; Supplementary Table S7); the PNI × MELD interaction was not individually significant in that smaller sample (*p* = 0.12) but pointed in the same direction. The stratified analysis is shown for the largest (MIMIC-IV) and most aetiologically distinct (institutional) cohorts; the eICU-CRD strata contained fewer deaths and were correspondingly less informative.

**Table 4:** Discrimination of PNI for 28-day mortality across MELD-defined severity strata (MIMIC-IV).

MELD stratum	Patients, *n*	Deaths, *n* (%)	PNI AUC (95% CI)
MELD < 15	593	98 (16.5)	0.602 (0.537–0.667)
MELD 15 to < 25	1,161	281 (24.2)	0.590 (0.550–0.629)
MELD ≥ 25	1,899	920 (48.4)	0.527 (0.501–0.553)
Overall	3,653	1,299 (35.6)	0.558 (0.538–0.578)

Discrimination (AUC) of PNI for 28-day mortality within prespecified MELD strata in the MIMIC-IV primary cohort (n = 3,653). PNI discrimination is weak in every stratum and lowest in the most severe stratum, with a point estimate close to chance; the stratum-specific confidence intervals overlap, so the apparent gradient should not be over-interpreted. The same stratified analysis reproduced this gradient in the institutional cohort (Supplementary Table S7). AUC, area under the receiver-operating-characteristic curve; other abbreviations as in Table 1.

Second, a fixed-weight composite such as PNI—whose albumin and lymphocyte terms are combined with the predetermined Onodera coefficients rather than weights optimized for this outcome—is not guaranteed to discriminate better than its most informative single component, and is diluted when one component carries little signal. The lymphocyte count that distinguishes PNI from albumin discriminated at or near chance in both cohorts (Supplementary Table S6): its univariable AUC was 0.512 (95% CI 0.492–0.532) in MIMIC-IV and 0.545 (0.501–0.589) in eICU-CRD—negligible in absolute terms, and inconsistent in that even the marginally above-chance eICU point estimate did not correspond to any independent association with mortality. Serum albumin was the more discriminative component in MIMIC-IV (AUC 0.578 versus 0.512), though only modestly and with an independent association confined to that cohort; in eICU-CRD the two components were comparable and both near chance (albumin 0.518, lymphocytes 0.545), neither independently associated with mortality. Adding a near-chance lymphocyte term to albumin under fixed Onodera weights therefore does not improve, and slightly degrades, discrimination relative to albumin alone. This aligns with the wider move in clinical nutrition away from albumin- and lymphocyte-based indices as stand-alone markers of nutritional status in acutely ill patients.

Two further analyses bear on these observations. First, measurement timing did not appear to explain the weak performance. Recomputing the PNI between 72 h and 7 days after ICU admission, once the acute phase had partly resolved, did not improve discrimination: within the 865 MIMIC-IV patients with both measurements the late PNI reached an AUC of 0.508 (95% CI 0.466–0.550) against 0.535 (0.494–0.576) for the baseline value (DeLong *p* = 0.18), and it was not independently associated with mortality after adjustment (odds ratio per SD 1.042, 0.899–1.209). The change in PNI between the two time points was itself uninformative (AUC 0.477, 0.435–0.518). The pattern was reproduced in eICU-CRD (428 paired patients; late PNI 0.470, 0.399–0.541, versus baseline 0.518, 0.446–0.591). This analysis conditions on survival and continued ICU stay to at least day 3, and discrimination of every marker including MELD was lower in this subgroup, so the two time points are compared with each other rather than with whole-cohort estimates (Supplementary Table S12).

Second, inflammatory markers behaved in front of MELD much as the nutritional index did. C-reactive protein was recorded within the ascertainment window for only 347 of 7,786 MIMIC-IV patients with liver disease (4.5%) and for approximately 4% of eICU-CRD admissions, and procalcitonin and interleukin-6 are not systematically available in either database, so this question could be addressed only in the institutional cohort, where both markers are measured routinely. There, C-reactive protein was available for 391 of 453 patients (86.3%) and procalcitonin for 308 (68.0%); the C-reactive protein subgroup reproduced the full-cohort estimates almost exactly (PNI AUC 0.547 versus 0.549; MELD 0.672 versus 0.674), indicating that it was not a biased subsample. C-reactive protein discriminated in-hospital mortality better than the PNI (0.608, 0.553–0.663, versus 0.547, 0.493–0.601) and, unlike the PNI, retained an independent association after adjustment for MELD, age and APACHE II (odds ratio per SD of the log-transformed value 1.234, 1.014–1.502, *p* = 0.036; 1.197, 0.992–1.444, *p* = 0.061 on the untransformed scale) whereas the PNI did not (0.935, 0.788–1.109, *p* = 0.44). Yet it added no more to MELD than the PNI did (ΔC +0.009, *p* = 0.28, versus +0.006, *p* = 0.44); procalcitonin behaved likewise (AUC 0.587; ΔC +0.008, *p* = 0.36). Within the index, C-reactive protein correlated appreciably with serum albumin (Spearman ρ = −0.371) but only weakly with the lymphocyte count (−0.142), and with the PNI as a whole at −0.336; adjustment for log-transformed C-reactive protein moved the already non-significant PNI association further toward the null (0.981, 0.820–1.173). The correlations were modest in absolute terms, indicating that inflammation accounts for only part of the variation in the index (Supplementary Table S13).

### 3.5 Consistency across the three cohorts

The three cohorts differed substantially in origin and case-mix—two North American research databases (one single-center, one multi-center) and a Chinese single-center cohort in which hepatitis B was the leading cause of liver disease—spanning median serum albumin from 2.9 to 2.4 g/dL and in-hospital mortality from 25.3% to 32.0%, yet the principal findings were concordant (Table 5). PNI discriminated in-hospital or 28-day mortality at 0.558, 0.520 and 0.549, uniformly close to chance, while MELD discriminated at 0.699, 0.696 and 0.674; MELD exceeded PNI by 0.141, 0.176 and 0.125 AUC units, significantly in every cohort. Adding PNI to MELD changed the C-statistic by no more than 0.005 and was non-significant in all three cohorts, and PNI was nearly uncorrelated with MELD throughout (r = −0.055 to −0.003). The weak adjusted PNI association seen in the MIMIC-IV primary cohort (OR 0.909, p = 0.014) reached significance in neither independent validation cohort (*p* = 0.95 and 0.38), whereas RDW retained an independent association in MIMIC-IV and the institutional cohort but not in eICU-CRD.

**Table 5:** Consistency of the principal findings across the three cohorts.

Measure	MIMIC-IV (*n* = 3,653)	eICU-CRD (*n* = 922)	Institutional (*n* = 453)
PNI AUC	0.558	0.520	0.549
MELD AUC	0.699	0.696	0.674
ΔAUC (MELD – PNI)	0.141	0.176	0.125
MELD + PNI, ΔC-statistic (DeLong *p*)	+0.003 (0.06)	−0.001 (0.71)	+0.005 (0.48)
PNI–MELD correlation, r	−0.055	−0.003	−0.038
PNI adjusted OR per SD (*p*)	0.909 (0.014)	1.006 (0.95)	0.928 (0.38)
Serum albumin adjusted OR per SD (*p*)	0.830 (< 0.001)	1.067 (0.44)	0.896 (0.21)
RDW adjusted OR per SD (*p*)	1.220 (< 0.001)	1.049 (0.58)	1.261 (0.011)

Summary of the headline metrics reported in Tables 2 and 3. Outcomes are 28-day mortality (MIMIC-IV) and in-hospital mortality (eICU-CRD and the institutional cohort). AUCs are DeLong estimates; ΔC is the change in the C-statistic on adding PNI to MELD, with the DeLong p for the difference; adjusted odds ratios are per one–standard-deviation increment from models including MELD, age and the cohort-specific severity score. Abbreviations as in Table 1.

That the three cohorts do not align perfectly is itself informative. MELD's absolute discrimination was somewhat lower in the institutional cohort (AUC 0.674) than in the two North American cohorts (0.699 and 0.696), plausibly reflecting the different pathophysiology of hepatitis-B-related decompensation and reported differences in creatinine generation across populations; even so, MELD remained far superior to PNI in that cohort. The two North American databases inevitably share features of population and practice, so the most distinct external test is the hepatitis-B-predominant institutional cohort; even so, the systematic differences in etiology, serum albumin and mortality across the three datasets indicate genuinely independent populations rather than re-analyses of a single source, which strengthens rather than weakens the central, reproduced result: in critically ill liver disease, PNI discriminates close to chance, does not outperform its own albumin component, and adds no clinically meaningful information beyond MELD.

## 4 Discussion

This study set out to test, rather than assume, the prognostic value of a widely used nutritional index, and to do so against the comparators and with the analyses that decide whether a marker is clinically useful. The answer was consistent across three independent cohorts of critically ill adults with liver disease, and it was negative: PNI discriminated short-term mortality only marginally better than chance, did not outperform serum albumin alone, and added essentially nothing to MELD. The lymphocyte term that distinguishes PNI from albumin carried little or no independent prognostic information. We read these results not as a dismissal of nutritional assessment, which remains clinically important in liver disease, but as evidence that this particular index does not deliver the prognostic information its widespread use presumes.

### 4.1 Comparison with previous studies

These findings stand in apparent contrast to a sizeable literature reporting that a low PNI predicts adverse outcomes across cancers (7, 8) and in chronic liver disease, including a report in decompensated cirrhosis in which PNI showed predictive power comparable to MELD (32). More recently, a low admission PNI has been associated with higher mortality across intensive-care cohorts of patients with sepsis (33); and PNI is only one of a broader family of composite indices derived from routine blood counts, of which inflammation-based scores such as the neutrophil-to-lymphocyte ratio show comparable associations with mortality in acute-on-chronic liver failure (34). Three considerations reconcile that literature with the present results. First, most prior studies evaluated PNI as an isolated exposure and reported its association with outcome rather than its discrimination or its incremental value over an established model such as MELD (13); a statistically significant odds ratio need not translate into useful discrimination or reclassification (28). Second, few studies separated the contribution of serum albumin from that of the lymphocyte count, so an apparent PNI signal may largely reflect its albumin component. Serum albumin is itself a potent, dose-dependent predictor of outcome in acute illness—an association that a large meta-analysis found to be largely independent of both nutritional status and inflammation (35), consistent with hypoalbuminaemia behaving as a marker of illness severity rather than of nutrition (9). The lymphocyte term fares no better as a nutritional marker: lymphopenia in critical illness arises chiefly from the acute stress response and from cirrhosis-associated immune dysfunction, and predicts mortality as a feature of that physiology rather than of undernutrition (10, 36). Third, the PNI evidence base is dominated by retrospective studies of generally low or very low certainty and is vulnerable to selective reporting, as a recent umbrella review emphasized (7). Prior positive reports and the present null discrimination are therefore not necessarily in conflict; they describe different quantities—association versus added predictive value—and the contribution here is to supply the comparative, incremental-value evidence that this earlier literature largely lacked.

### 4.2 Why a routine red-cell index outperformed the nutritional score

One secondary observation deserves comment. RDW—a routinely reported index of red-cell size variation linked to systemic inflammation and oxidative stress—out-discriminated PNI in all three cohorts and retained an independent association with mortality in two of them. This is consistent with reports that elevated RDW predicts short-term mortality in decompensated cirrhosis (37) and, more broadly, across general critically ill populations, a systematic review having linked higher RDW to increased mortality in non-cardiovascular acute illness (38). We read this not as a recommendation to adopt RDW, whose discrimination here was itself only modest and which added little to MELD, but as an illustration that the weak performance of PNI is a property of that specific index rather than of inexpensive hematological markers in general. A parallel observation applies to BAR (the blood urea nitrogen–to–albumin ratio), which likewise out-discriminated PNI in all three cohorts (Table 2); because BAR is built from urea—a marker of renal dysfunction and catabolism—and albumin, both of which track illness severity rather than nutritional state, its advantage over a nutrition-framed index is what the present account would predict, and it reinforces that the weakness is specific to PNI rather than to inexpensive composites as a class. These comparator analyses were exploratory rather than prespecified for confirmatory testing.

### 4.3 Clinical and research implications

Clinically, these results argue against using PNI as a prognostic instrument in critically ill liver disease, and in particular against presenting it as adding to MELD. For short-term mortality risk, MELD—including its recalibrated MELD 3.0 formulation—remains the appropriate basis for prognostication (11, 12). The more consequential implication, however, is for nutritional assessment itself. That PNI failed even as a discriminator, with what little signal it carried residing in albumin rather than in the lymphocyte term, is consistent with the account, drawn from prior work (9, 10), that both components are strongly influenced by systemic inflammation and chronic physiologic reserve rather than by nutritional state—the very confounding that the GLIM framework confronts in recognizing inflammation, alongside reduced intake and depleted reserves, as an aetiologic criterion for malnutrition (4). Because the present data speak to prognostic discrimination rather than to nutritional status directly, they do not by themselves establish that the PNI fails as a nutritional marker; but a fixed-weight blood-based index that conflates nutrition with inflammation is unlikely to substitute for criterion-based nutritional assessment, which current guidelines anchor instead to tools validated for that purpose. Liver-specific screening instruments such as the Royal Free Hospital-Nutritional Prioritizing Tool, which independently predicts deterioration of liver function and survival in cirrhosis (39), are designed for this task in a way that an albumin-based composite is not. Where the question is muscle and functional status rather than screening, direct assessment is both feasible and more informative: sarcopenia is a biologically grounded predictor of mortality in cirrhosis (40) and, in contrast to the PNI, has been reported to add incremental value to MELD—for example through the MELD-Sarcopenia score (41) and computed-tomography measures of muscle such as psoas thickness (42)—although these comparisons were not tested in the present study. Frailty, captured by simple bedside instruments including the Liver Frailty Index (5) and the Clinical Frailty Scale (43), complements these measures and likewise predicts hospitalization and death. The recurring theme is that the markers which add information to MELD are those that measure something MELD omits—muscle and physical function—rather than a rescaling of two variables already shaped by illness severity. More broadly, the distinction between statistical discrimination and clinical usefulness means that a marker earns a place in practice only by improving on the information already available to the clinician (44); because PNI added essentially nothing to the discrimination or risk classification already provided by MELD, it cannot improve on decisions that are based on MELD.

The inflammatory-marker data from the institutional cohort sharpen this reading. Inflammation was not prognostically inert here: C-reactive protein discriminated better than the PNI and, unlike the PNI or albumin, remained independently associated with mortality after adjustment for severity on the log scale (*p* = 0.036; *p* = 0.061 untransformed). What it did not do was add to MELD. Neither a canonical inflammatory marker nor a fixed-weight nutritional index added meaningfully to a disease-specific organ-failure score in this cohort, and the component of the PNI more closely related to inflammation—albumin—was also the component carrying what little discrimination the index possesses. These observations are consistent with the interpretation that the PNI functions in critical illness as a weak, non-specific marker of inflammation and physiologic reserve rather than of nutritional status, without establishing it; they also locate a more general point, that in decompensated critical illness the prognostic information carried by the circulating markers examined here is largely already contained in the organ-failure score. A related question is whether the index is simply being asked to perform in the wrong window, since it was devised to grade nutritional risk before elective surgery rather than during acute illness; re-measurement between days 3 and 7 did not support this in an exploratory paired analysis restricted to patients surviving to day 3, in which discrimination did not improve once the acute phase had partly resolved. These findings should not, however, be extrapolated beyond the setting in which they were obtained. In stable outpatients with chronic liver disease the distribution of the PNI is wider and the acute perturbations that dominate here are absent, so prospective validation in non-acute and ambulatory settings, with concurrent criterion-based nutritional assessment and body-composition measurement, remains warranted.

Why an albumin-based index should add so little to a contemporary MELD follows from how the two scores are built rather than from the particular data analyzed here. What little prognostic signal the PNI carries resides in its albumin term and not in its lymphocyte term, and MELD 3.0 already contains serum albumin as a weighted component, with that weight estimated in a liver-disease population rather than fixed *a priori* as in the Onodera formula. A clinician using MELD 3.0 has therefore already extracted the prognostic content of albumin, and an albumin-based nutritional index can contribute only its lymphocyte term, which discriminated at or near chance in both public cohorts. That the incremental value of the PNI was smallest over MELD 3.0 in MIMIC-IV is what this account predicts; in eICU-CRD every increment was indistinguishable from zero, so the ordering there carries no information either way (Supplementary Table S10).

### 4.4 Strengths and limitations

This study has several strengths. It drew on three independent cohorts spanning different health systems, etiologies and case-mix, combining a large primary database with both external and institutional validation, and it applied a prespecified evaluation framework that moved deliberately beyond association to discrimination, reclassification and incremental value (13, 15, 28). That approach reflects the now-standard view that a candidate marker must be judged by its incremental contribution over an established model rather than by association alone (45); we centered the appraisal on discrimination and risk reclassification, the quantities that directly answer whether PNI adds prognostic information beyond MELD. A decision-curve analysis (Supplementary Table S9) confirmed that adding the PNI to MELD yielded no material improvement in net benefit across the clinically relevant range of decision thresholds, consistent with the near-zero change in C-statistic and integrated discrimination improvement, neither of which was reproduced in external validation; formal calibration would further characterize a marker that did show incremental value but was not required to establish the present null. The appraisal was complemented by sensitivity analyses including E-values for unmeasured confounding (30). Several limitations should temper interpretation, and follow in part from the data sources. The analyses were retrospective and observational, so residual confounding cannot be excluded despite covariate adjustment and E-value analysis (30). Two cohorts were drawn from large critical-care databases (16, 17); such datasets afford scale and granularity but rely on routinely recorded laboratory values, taken for the principal analyses at a single early time point (with re-measurement between days 3 and 7 examined separately, Supplementary Table S12), and are subject to variation in measurement timing, coding and missingness; neither database records body composition or serial nutritional assessment, which would be required to compare the PNI directly against a validated nutritional instrument. The primary analyses were complete-case, excluding 53.1% of the MIMIC-IV, 58.2% of the eICU-CRD and 28.9% of the institutional source populations, and the institutional cohort was single-center, so selection effects cannot be excluded. Missingness was driven predominantly by the lymphocyte differential rather than by albumin, reflecting that a differential count is ordered selectively whereas albumin belongs to routine chemistry; analyzed patients were, if anything, more severely ill than those excluded, and multiple imputation across the full source populations left the adjusted association unchanged in direction and magnitude in MIMIC-IV (odds ratio per SD 0.907, 0.847–0.971, against 0.909, 0.842–0.981) and moved it slightly below unity in eICU-CRD (0.932, 0.819–1.060, against 1.006, 0.855–1.183), where the imputed area under the curve was also somewhat higher than the complete-case value (0.554 against 0.520); both associations remained non-significant and discrimination remained close to chance on both analyses (Supplementary Table S11). The concordance of findings across cohorts of differing case-mix likewise makes a spurious shared result unlikely. Because database constraints required a different severity score in each cohort (SAPS-II, APACHE IV and APACHE II), the adjusted models are not identical across cohorts, so the cross-cohort concordance is best read as reproduction of the same qualitative finding rather than as strictly homogeneous replication. No criterion-based nutritional instrument—NRS-2002, the Royal Free Hospital-Nutritional Prioritizing Tool, GLIM criteria, body composition or performance-based frailty—was available in retrievable form in any cohort: the two public databases do not record them, and in the institutional cohort nutritional screening is documented in free-text nursing records that could not be extracted retrospectively. These tools could therefore not be evaluated head-to-head against the PNI, and their advantage in this specific setting is inferred from prior validation rather than demonstrated here; inflammatory markers were measured in only one of the three cohorts, and serum sodium was not retrieved for the institutional cohort, so MELD-Na and MELD 3.0 could be examined only in the two public cohorts. Finally, we assessed short-term mortality only and cannot exclude that PNI carries information for other outcomes, longer horizons or non-critically-ill populations not examined here; and because body composition was not measured directly, our results speak to PNI as a prognostic discriminator rather than to whether its components adequately capture nutritional status—a distinction that itself argues for direct nutritional assessment.

## 5 Conclusion

In three independent cohorts of critically ill adults with liver disease, the Prognostic Nutritional Index discriminated short-term mortality only marginally above chance, did not outperform serum albumin alone, and added no clinically meaningful information beyond MELD; the lymphocyte term that distinguishes PNI from albumin carried essentially no independent prognostic signal. Because these findings were reproduced across populations differing in geography, etiology and case-mix—including an independent institutional cohort with a markedly different, hepatitis-B-predominant case-mix—they are unlikely to be artifacts of a single setting. These findings therefore argue against relying on the PNI for mortality risk stratification in critically ill liver disease. They apply to short-term mortality in critically ill patients, in whom the PNI distribution is compressed and largely below its conventional threshold; they do not address its performance for other outcomes, over longer horizons, or in less severely ill or ambulatory populations, in whom its distribution is wider. This is a constructive conclusion as much as a cautionary one: because the index appears to track systemic inflammation and chronic physiologic reserve—a pattern consistent with prior work and, for its albumin component, with the inflammatory-marker data reported here—genuine nutritional risk is better identified by validated, guideline-concordant assessment of malnutrition, sarcopenia and frailty—measures that quantify what they intend to and, unlike a rescaled albumin–lymphocyte index, add to what MELD already provides—while short-term mortality risk itself remains well served by established disease-specific models such as MELD or MELD 3.0.

## Data Availability

The datasets presented in this study can be found in online repositories. MIMIC-IV and eICU-CRD are available to credentialed users through the PhysioNet repository (https://physionet.org/) upon completion of the required training and data-use agreement, and the analysis code and SQL queries are available in the Zenodo repository (https://doi.org/10.5281/zenodo.20894993). The institutional dataset analyzed in this article is not readily available because of patient-privacy and ethical restrictions; requests to access it should be directed to the corresponding author.
