# Supplementary material for: The prognostic nutritional index vs. MELD for short-term mortality in critically ill liver disease: a retrospective multi-cohort study
Source: Front Nutr. 2026 Sep 7;13:1930417. doi: 10.3389/fnut.2026.1930417 (PMC13590390; doi:10.3389/fnut.2026.1930417)
Supplement: Supplementary file 1 [file Table_1.docx]

**Supplementary Material**

This appendix provides the cross-cohort variable and unit specification, sensitivity analyses in the wider marker-available populations, a comparison of complete and incomplete cases, data-integrity checks, E-values for the adjusted associations, the univariable discrimination of PNI’s individual components, additional institutional-cohort analyses (complete-versus-excluded comparison, aetiology and severity-stratified discrimination), an exploratory note on red-cell distribution width, a decision-curve analysis, comparisons against MELD-Na and MELD 3.0, a multiple-imputation analysis across the full source populations, re-measurement of the PNI between days 3 and 7, the relationship of the index to directly measured inflammatory markers in the institutional cohort, and the rule used to ascertain the MELD components in MIMIC-IV. All estimates were recomputed from the source data with the same analytic pipeline used in the main text; the completed STROBE and TRIPOD-informed reporting checklists are provided as separate files at submission rather than reproduced here. Results for the institutional cohort are included throughout rather than deferred.

**Supplementary Table S1.** Cross-cohort variable definitions and unit harmonisation.

| **Variable** | **MIMIC-IV (source, unit)** | **eICU-CRD (source, unit)** | **Institutional (source)** | **Harmonised target and conversion** |
| --- | --- | --- | --- | --- |
| Serum albumin | albumin (g/dL) | albumin (g/dL) | HIS (g/L) | g/dL (g/L ÷ 10) |
| Lymphocyte count | absolute count (cells/µL) | absolute count (cells/µL) | HIS (10⁹/L) | cells/µL (10⁹/L × 1000) |
| Total bilirubin | mg/dL | mg/dL | HIS (µmol/L) | mg/dL (µmol/L ÷ 17.1) |
| Creatinine | mg/dL | mg/dL | HIS (µmol/L) | mg/dL (µmol/L ÷ 88.4); capped at 4.0 |
| INR | INR | INR | HIS (INR) | unitless; floored at 1.0 |
| RDW | % | % | HIS (%) | % (no conversion) |
| Blood urea nitrogen | mg/dL | mg/dL | HIS (urea, mmol/L) | mg/dL (urea mmol/L ÷ 0.357 if needed) |
| PNI | derived | derived | derived | 10 × albumin + 0.005 × lymphocytes |
| MELD | derived | derived | derived | 9.57·ln(creat) + 3.78·ln(bili) + 11.20·ln(INR) + 6.43 |
| BAR | derived | derived | derived | BUN(mg/dL) ÷ albumin(g/dL) |
| Severity score | SAPS-II | APACHE IV | APACHE II | cohort-specific score |
| Liver disease | ICD-9/10 codes | diagnosis strings | ICD-10 codes | cirrhosis or acute/ACLF |
| Mortality | in-hospital, 28-/90-day | in-hospital, ICU | in-hospital | in-hospital common; longer-term where available |
| Serum sodium | chemistry (mEq/L) | lab: sodium (mmol/L) | not extracted | mmol/L; MELD-Na and MELD 3.0 only |
| C-reactive protein | inflammation (mg/L); recorded in 4.5% | lab: CRP; recorded in ≈4% | HIS (mg/L); recorded in 86.3% | mg/L (no conversion) |
| Procalcitonin | not systematically recorded | not systematically recorded | HIS (ng/mL); recorded in 68.0% | ng/mL (no conversion) |
| Ascertainment window | −6 h to +72 h from ICU admission (MELD: most abnormal value on ICU day 1) | −6 h to +72 h from ICU admission | 0 to +72 h from ICU admission | first available value in the stated window |

***Sensitivity analyses in wider marker-available populations***

Because the main analyses were restricted to complete cases, each marker was re-examined in the wider population in which that marker (with MELD and a severity score) was available, without requiring the other markers. The conclusions were unchanged. In MIMIC-IV, serum albumin retained an independent inverse association in the albumin-available population (n = 5,975; adjusted per-SD OR 0.841, 95% CI 0.791–0.894), and RDW retained an independent positive association in the RDW-available population (n = 7,701; adjusted OR 1.204, 1.138–1.274); MELD discrimination was essentially identical to the main analysis (AUC 0.699 in the full extraction of 7,786 patients). In eICU-CRD, PNI discrimination remained poor in the larger PNI-available population (n = 1,331; AUC 0.548) while MELD again discriminated well (AUC 0.701, computed in the PNI- and MELD-complete subset of 1,043 patients; Table S2), and the borderline univariable PNI association (OR 0.874, 0.763–1.001, p = 0.05) did not persist in the complete-case population, in which the unadjusted estimate was already null. In the institutional cohort the same widening left the conclusions unchanged: MELD discriminated at 0.672 in the APACHE-available population (n = 581), essentially identical to its main-analysis value, and RDW retained an independent association (n = 564; adjusted per-SD OR 1.243, p = 0.005), whereas albumin did not (n = 531; 0.903, p = 0.17). Estimates are summarised in Table S2.

**Supplementary Table S2.** Marker performance in wider marker-available populations.

| **Cohort** | **Marker (population)** | **n (events)** | **AUC (95% CI)** | **Adjusted per-SD OR (95% CI)** |
| --- | --- | --- | --- | --- |
| MIMIC-IV | Albumin (albumin-available) | 5,975 (1,922) | 0.573 (0.557–0.589) | 0.841 (0.791–0.894), p < 0.001 |
|  | RDW (RDW-available) | 7,701 (2,457) | 0.584 (0.570–0.598) | 1.204 (1.138–1.274), p < 0.001 |
|  | MELD (full extraction) | 7,786 (2,513) | 0.699 (0.687–0.712) | — |
| eICU-CRD | PNI (PNI-available) | 1,331 (292) | 0.548 (0.508–0.587) | 0.874 (0.763–1.001)†, p = 0.05 |
|  | MELD (PNI- and MELD-complete subset) | 1,043 (254) | 0.701 (0.666–0.737) | — |
|  | RDW (RDW-complete) | 1,183 (319) | 0.576 (0.540–0.612) | 1.073 (0.921–1.251), p = 0.36 |
| Institutional | Albumin (+MELD+APACHE) | 531 (149) | 0.551 (0.505–0.597) | 0.903 (0.782–1.043), p = 0.17 |
|  | RDW (+MELD+APACHE) | 564 (158) | 0.594 (0.549–0.639) | 1.243 (1.068–1.446), p = 0.005 |
|  | MELD (+APACHE) | 581 (162) | 0.672 (0.635–0.709) | — |

*† Unadjusted per-SD odds ratio, computed in the PNI-available population of eICU-CRD (n = 1,331); all other estimates in this table are adjusted for MELD, age and the cohort-specific severity score. Abbreviations as in the main text.*

***Comparison of complete and incomplete cases***

In MIMIC-IV, the 3,653 patients with a measurable PNI differed modestly from the 4,133 without one (Table S3). Patients with a complete PNI had a higher median MELD score (25 vs 22) and higher 28-day (35.6% vs 29.4%) and in-hospital (32.0% vs 25.7%) mortality, whereas the incomplete-case group was slightly older and contained a higher proportion with cirrhosis. The complete-case sample was therefore, if anything, more severely ill; together with the wider-subset analyses (Table S2), in which the same conclusions held, this argues against complete-case restriction having produced a spuriously poor PNI performance. In eICU-CRD, the primary-analysis population (n = 922) and the remainder (n = 1,284) had similar ages (median 56 vs 58 years) and in-hospital mortality (25.3% vs 22.5%); on re-examination for this revision, the PNI was the most frequent single reason for exclusion (missing in 875 of the 1,284 patients not analysed, 68.1%), with a further 409 excluded for a missing severity score or MELD component despite an available PNI; the MELD summarised above for the excluded group is that of the 524 patients in whom it could be computed. In the institutional cohort the 453 analysed patients were, similarly, somewhat more severely ill than the 184 excluded (median MELD 22 vs 19, in-hospital mortality 29.1% vs 23.9%; Table S4), so complete-case restriction was again, if anything, conservative.

**Supplementary Table S3.** Characteristics of complete versus incomplete cases.

| **Characteristic** | **MIMIC-IV, PNI complete (n = 3,653)** | **MIMIC-IV, PNI missing (n = 4,133)** | **eICU-CRD complete (n = 922)** | **eICU-CRD incomplete (n = 1,284)** |
| --- | --- | --- | --- | --- |
| Age, years | 60 (51–69) | 62 (53–71) | 56 (49–64) | 58 (50–66) |
| Male sex, % | 61.8 | 63.0 | 61.7 | 60.2 |
| Cirrhosis, % | 66.4 | 75.7 | 73.6 | 76.1 |
| MELD score | 25 (18–33) | 22 (14–29) | 21 (16–29) | 22 (15–31)* |
| In-hospital death, % | 32.0 | 25.7 | 25.3 | 22.5 |
| 28-day death, % | 35.6 | 29.4 | — | — |

**Supplementary Table S4.** Institutional cohort: analysed versus excluded cases.

| **Characteristic** | **Analysed (n = 453)** | **Excluded (n = 184)** |
| --- | --- | --- |
| Age, years | 55 (46–65) | 58 (48–67) |
| Male sex, % | 65.3 | 63.0 |
| Cirrhosis, % | 76.2 | 73.4 |
| MELD score | 22 (16–30) | 19 (13–26) |
| In-hospital death, % | 29.1 | 23.9 |

***Data-integrity checks***

Three prespecified integrity checks were performed before analysis. First, the PNI values supplied in each cohort were reproduced from their albumin and lymphocyte inputs using the Onodera formula; the maximum absolute discrepancy was 1.4 × 10^-14^ in both cohorts, confirming that PNI had been computed on the intended scale. Second, the lymphocyte counts were confirmed to be on an absolute (cells/µL) rather than a percentage scale: the median was 890 cells/µL in MIMIC-IV and 881 cells/µL in eICU-CRD in the full marker-available extraction (Table 1 reports the analysis-cohort medians of 880 and 878), with approximately 97–99% of values falling within the plausible range of 100–10,000 cells/µL (about 97.5% in MIMIC-IV and 98.5% in eICU-CRD); the remaining 1–3% outside this range was truncated to the range bounds before PNI was computed (Methods). Third, each cohort contained one record per patient, with no duplicated stay identifiers (0 duplicates in either cohort). The MELD score was recomputed with laboratory values floored at 1.0 and creatinine capped at 4.0 mg/dL, per convention. The same checks in the institutional cohort gave a maximum PNI-reproduction discrepancy below 10^-13^, a median lymphocyte count of 810 cells/µL with 96.7% of values within the plausible range (15 values [3.3%] truncated to the bounds), and no duplicated ICU records.

***E-values for the adjusted associations***

E-values quantify the minimum strength of association, on the risk-ratio scale, that an unmeasured confounder would need to have with both the marker and mortality to fully explain an observed adjusted association. Because the 28-day outcome is common, the risk ratio was approximated from the adjusted odds ratio before computing each E-value. On this scale the adjusted MIMIC-IV associations were modest and would require only a moderately strong unmeasured confounder to overturn (Table S5); the weak adjusted PNI association has the smallest E-value (1.28; lower confidence bound 1.11), meaning that a relatively modest unmeasured confounder could account for it.

**Supplementary Table S5.** E-values for adjusted per-SD associations (MIMIC-IV, 28-day mortality).

| **Marker** | **Adjusted OR (95% CI)** | **E-value (point)** | **E-value (CI bound)** |
| --- | --- | --- | --- |
| RDW | 1.220 (1.127–1.321) | 1.44 | 1.32 |
| Serum albumin | 0.830 (0.768–0.896) | 1.43 | 1.30 |
| PNI | 0.909 (0.842–0.981) | 1.28 | 1.11 |

***Univariable discrimination of PNI’s components***

PNI is a weighted sum of serum albumin and the peripheral lymphocyte count combined with the fixed Onodera coefficients; such a fixed-weight composite is not guaranteed to discriminate better than its most informative component, and is diluted when one component carries little signal. Table S6 reports the univariable discrimination of each component for the primary outcome of each cohort. The peripheral lymphocyte count discriminated at or near chance in both cohorts (AUC 0.512 in MIMIC-IV, 0.545 in eICU-CRD), whereas serum albumin carried the more substantial — though still modest — discriminative signal (0.578 and 0.518). Because PNI combines a near-chance lymphocyte term with albumin under fixed weights, its discrimination does not exceed that of albumin alone in either cohort, consistent with the head-to-head comparison in the main text. A variance decomposition is not reported, because the variance of PNI in MIMIC-IV is sensitive to a small number of extreme lymphocyte counts; after truncation to 100–10,000 cells/µL the variance is dominated by albumin in both cohorts.

**Supplementary Table S6.** Univariable discrimination of PNI and its components.

| **Cohort** | **Index or component** | **Univariable AUC (95% CI)** |
| --- | --- | --- |
| MIMIC-IV | Prognostic Nutritional Index | 0.558 (0.538–0.578) |
|  | Serum albumin | 0.578 (0.558–0.598) |
|  | Lymphocyte count | 0.512 (0.492–0.532) |
| eICU-CRD | Prognostic Nutritional Index | 0.520 (0.475–0.565) |
|  | Serum albumin | 0.518 (0.474–0.561) |
|  | Lymphocyte count | 0.545 (0.501–0.589) |

***Exploratory note on red-cell distribution width***

Among the candidate markers, RDW showed the strongest independent association with mortality in the MIMIC-IV primary cohort (adjusted per-SD OR 1.220, 95% CI 1.127–1.321, p < 0.001), persisting after adjustment for MELD, age and severity. This independent signal did not replicate in eICU-CRD: although RDW was associated with in-hospital mortality on univariable analysis (per-SD OR 1.209 in the primary population and 1.264 in the wider RDW-complete population, both p < 0.05), the association was abolished after adjustment (adjusted OR 1.049, p = 0.58 in the primary population; 1.073, p = 0.36 in the wider population). RDW is therefore reported as an exploratory comparator only; its apparent independence in the primary cohort should be regarded as hypothesis-generating, and one plausible interpretation is that in the validation cohort the prognostic information carried by RDW was already captured by the APACHE IV severity score. This contrast is itself a reminder that single-cohort marker associations require external confirmation — the same caution that motivates the present appraisal of PNI.

***Institutional cohort: aetiology and severity-stratified discrimination***

The institutional cohort provides validation in a distinct case-mix. Among the 345 patients with cirrhosis, the leading aetiology was chronic hepatitis B (48.1%), followed by alcohol-related liver disease (24.9%), with hepatitis C, autoimmune/cholestatic and mixed or cryptogenic causes making up the remainder (Table S8) — a profile that contrasts with the alcohol-predominant North American cohorts and reflects the intermediate-to-high hepatitis B prevalence of the region; hepatocellular carcinoma or other malignancy was present in 18.8% of the cohort. The number of patients entering analysis was stable across the seven calendar years (annual in-hospital mortality 23.5–33.8%, χ² p = 0.69), arguing against secular drift. Severity-stratified analysis reproduced the primary-cohort gradient: PNI discrimination declined monotonically from mild to severe disease and was indistinguishable from chance in the most severe stratum (Table S7), where most deaths occurred. The PNI × MELD interaction was not individually significant in this smaller sample (p = 0.12) but pointed in the same direction as in MIMIC-IV.

**Supplementary Table S7.** Institutional cohort: discrimination of PNI for in-hospital mortality across MELD-defined severity strata.

| **MELD stratum** | **Patients, n** | **Deaths, n (%)** | **PNI AUC (95% CI)** |
| --- | --- | --- | --- |
| MELD < 15 | 94 | 14 (14.9) | 0.610 (0.420–0.800) |
| MELD 15 to < 25 | 175 | 40 (22.9) | 0.568 (0.468–0.668) |
| MELD ≥ 25 | 184 | 78 (42.4) | 0.527 (0.453–0.601) |
| Overall | 453 | 132 (29.1) | 0.549 (0.498–0.600) |

**Supplementary Table S8.** Institutional cohort: aetiology of cirrhosis.

| **Aetiology** | **n** | **%** |
| --- | --- | --- |
| Chronic hepatitis B | 166 | 48.1 |
| Alcohol-related | 86 | 24.9 |
| Chronic hepatitis C | 24 | 7.0 |
| Autoimmune / cholestatic | 17 | 4.9 |
| Mixed / other / cryptogenic | 52 | 15.1 |

**Supplementary Table S9.** Decision-curve analysis: change in net benefit (ΔNB) from adding PNI to MELD across decision thresholds.

| **Decision threshold** | **MIMIC-IV: ΔNB (MELD + PNI − MELD)** | **eICU-CRD: ΔNB (MELD + PNI − MELD)** |
| --- | --- | --- |
| 20% | +0.0023 | −0.0008 |
| 30% | +0.0022 | +0.0042 |
| 40% | −0.0017 | −0.0011 |
| 50% | +0.0060 | −0.0011 |
| 60% | −0.0031 | +0.0000 |
| Maximum \|ΔNB\| (thresholds 5–90%) | 0.0118 | 0.0081 |

*ΔNB is the difference in net benefit between a logistic model containing MELD + PNI and one containing MELD alone (28-day mortality in MIMIC-IV; in-hospital mortality in eICU-CRD); positive values favour MELD + PNI. Across the full 0.05–0.90 range the two strategies were near-identical: adding the PNI shifted net benefit by at most about 0.01 in either cohort, with no consistent direction, so the MELD + PNI curve was effectively superimposed on MELD and conferred no material advantage; the treat-all and treat-none strategies were inferior at all thresholds above the disease prevalence. Net benefit = (true positives/n) – (false positives/n) × [pt/(1–pt)], where pt is the threshold probability. Abbreviations as in the main text.*

**Further sensitivity analyses**

The five analyses that follow examine the PNI against two contemporary MELD variants, the robustness of the findings to the proportion of patients excluded for missing data, the behaviour of the index when re-measured after the acute phase, and its relationship to directly measured inflammatory markers in the institutional cohort. The last addresses the rule used to ascertain the MELD components in MIMIC-IV.

**Supplementary Table S10. Discrimination and incremental value of the PNI relative to MELD, MELD-Na and MELD 3.0.**

| **Cohort** | **Reference** | **n** | **AUC of reference (95% CI)** | **ΔAUC vs PNI (p)** | **C (ref)** | **C (ref+PNI)** | **ΔC (p)** |
| --- | --- | --- | --- | --- | --- | --- | --- |
| MIMIC-IV (28-day mortality) | MELD | 3,653 | 0.699 (0.681–0.717) | +0.141 (1.7×10⁻²⁴) | 0.6990 | 0.7025 | +0.0035 (0.06) |
|  | MELD-Na | 3,653 | 0.694 (0.676–0.712) | +0.136 (6.0×10⁻²³) | 0.6939 | 0.6970 | +0.0031 (0.09) |
|  | MELD 3.0 | 3,516 | 0.696 (0.678–0.714) | +0.140 (4.0×10⁻²⁴) | 0.6964 | 0.6981 | +0.0017 (0.29) |
| eICU-CRD (in-hospital mortality) | MELD | 922 | 0.696 (0.658–0.734) | +0.176 (7.2×10⁻⁹) | 0.6960 | 0.6954 | −0.0006 (0.71) |
|  | MELD-Na | 922 | 0.689 (0.651–0.727) | +0.169 (2.2×10⁻⁸) | 0.6891 | 0.6890 | −0.00005 (0.96) |
|  | MELD 3.0 | 922 | 0.663 (0.623–0.702) | +0.143 (1.7×10⁻⁶) | 0.6626 | 0.6630 | +0.0004 (0.50) |

*Areas under the receiver-operating-characteristic curve with DeLong 95% confidence intervals; pairwise comparisons and changes in the C-statistic were tested by the DeLong method for correlated curves. MELD-Na applies the standard sodium correction when MELD exceeds 11, with sodium bounded to 125–137 mmol/L; MELD 3.0 uses the published coefficients with creatinine bounded to 1.0–3.0 mg/dL, albumin to 1.5–3.5 g/dL and sodium to 125–137 mmol/L; both scores were truncated to 6–40. To keep the three scores comparable, all were derived from the same laboratory inputs. Serum sodium was available for 3,653/3,653 MIMIC-IV and 922/922 analytic patients; MELD 3.0 requires the individual score components; because the derived MELD concept substitutes the conventional lower bound when a component is absent, a MELD value exists for some patients in whom bilirubin (96) or the international normalised ratio (53) was not recorded (overlapping categories), reducing the MIMIC-IV denominator for MELD 3.0 to 3,516. Within that subset the PNI reached an AUC of 0.557, which is the comparator used for the corresponding ΔAUC. In eICU-CRD the MELD 3.0 components were re-extracted for this analysis and the recomputed MELD agreed closely with the score used in the main analysis (r = 0.96). Abbreviations as in the main text.*

Because complete-case restriction removed a substantial proportion of both public source populations, the marker analyses were repeated after multiple imputation across the entire source populations rather than the analysed subsets.

**Supplementary Table S11. Multiple-imputation sensitivity analysis in the full source populations.**

| **Cohort** | **Analysis** | **n** | **Adjusted PNI OR per SD (95% CI)** | **PNI AUC** |
| --- | --- | --- | --- | --- |
| MIMIC-IV | Complete case (main analysis) | 3,653 | 0.909 (0.842–0.981) | 0.558 (0.538–0.578) |
|  | Multiple imputation, m = 10 | 7,786 | 0.907 (0.847–0.971) | 0.555 (SD across imputations 0.005) |
| eICU-CRD | Complete case (main analysis) | 922 | 1.006 (0.855–1.183) | 0.520 (0.475–0.565) |
|  | Multiple imputation, m = 10 | 2,206 | 0.932 (0.819–1.060) | 0.554 (SD across imputations 0.006) |

*Multiple imputation by chained equations with ten imputations (van Buuren and Groothuis-Oudshoorn); the imputation model contained albumin, lymphocyte count, RDW, urea, MELD, the cohort-specific severity score, age, sex, diagnosis category (cirrhosis, liver failure), the outcome and, in MIMIC-IV only, the Charlson Comorbidity Index (not recorded in eICU-CRD), and the PNI was recomputed after imputation. Odds ratios are per one–standard-deviation increment, adjusted for MELD, age and the cohort-specific severity score, and pooled by Rubin’s rules. Missingness in the source populations was driven mainly by the lymphocyte differential (43.2% in MIMIC-IV, 32.3% in eICU-CRD) rather than by albumin (23.3% and 15.1%), reflecting that a differential count is ordered selectively whereas albumin belongs to routine chemistry. Abbreviations as in the main text.*

The PNI was devised to grade nutritional risk before elective surgery. To test whether it carries different prognostic information once the acute phase has partly resolved, it was recomputed from laboratory values obtained between 72 hours and 7 days after ICU admission and compared with the baseline value in the same patients.

**Supplementary Table S12. PNI re-measured between days 3 and 7, compared with the baseline value within the same patients.**

| **Cohort** | **Marker** | **n** | **AUC (95% CI)** |
| --- | --- | --- | --- |
| MIMIC-IV (28-day mortality) | Baseline PNI | 865 | 0.535 (0.494–0.576) |
|  | PNI, days 3–7 | 865 | 0.508 (0.466–0.550) |
|  | Baseline serum albumin | 865 | 0.523 (0.482–0.564) |
|  | Serum albumin, days 3–7 | 865 | 0.495 (0.454–0.537) |
|  | Change in PNI (late − baseline) | 865 | 0.477 (0.435–0.518) |
|  | MELD | 865 | 0.590 (0.551–0.630) |
| eICU-CRD (in-hospital mortality) | Baseline PNI | 428 | 0.518 (0.446–0.591) |
|  | PNI, days 3–7 | 428 | 0.470 (0.399–0.541) |
|  | Baseline serum albumin | 428 | 0.505 (0.437–0.573) |
|  | Serum albumin, days 3–7 | 428 | 0.464 (0.392–0.536) |
|  | MELD | 428 | 0.638 (0.578–0.699) |

*The late PNI was computed from the first albumin and lymphocyte values obtained between 72 hours and 7 days after ICU admission (median sampling time in MIMIC-IV 99 hours, IQR 82–127). Comparisons are restricted to patients with both measurements so that case-mix is held constant. Late versus baseline PNI: ΔAUC −0.027 (DeLong p = 0.18) in MIMIC-IV and −0.048 (p = 0.13) in eICU-CRD. After adjustment for MELD, age and the cohort-specific severity score the late PNI was not associated with mortality (OR per SD 1.042, 0.899–1.209, p = 0.58 in MIMIC-IV; 1.007, 0.792–1.281, p = 0.95 in eICU-CRD). This analysis conditions on survival and continued ICU stay to at least day 3: paired patients had a longer ICU stay (median 6.0 versus 2.6 days) and more sepsis (62.4% versus 55.3%) than unpaired patients, although 28-day mortality was similar (35.3% versus 35.7%), and discrimination of all markers including MELD was lower in this subgroup. The analysis is exploratory. Abbreviations as in the main text.*

C-reactive protein and procalcitonin are measured routinely in the institutional cohort but not in the two public databases, allowing the relationship between the PNI, systemic inflammation and mortality to be examined directly in one of the three cohorts.

**Supplementary Table S13. Inflammatory markers, the PNI and MELD in the institutional cohort.**

**Panel A. Availability and distribution**

|  | **C-reactive protein** | **Procalcitonin** |
| --- | --- | --- |
| Available, n (% of 453) | 391 (86.3) | 308 (68.0) |
| Deaths in analysed subgroup, n (%) | 113 (28.9) | 94 (30.5) |
| Median (IQR) | 23.8 mg/L (9.4–51.6) | 1.86 ng/mL (0.38–6.42) |
| Survivors, median (IQR) | 19.5 (7.8–43.2) | 1.43 (0.32–5.18) |
| Non-survivors, median (IQR) | 34.1 (14.6–68.3) | 3.24 (0.62–9.87) |
| Mann–Whitney p | 0.002 | 0.011 |

**Panel B. Representativeness of the C-reactive protein subgroup**

|  | **Full institutional cohort (n = 453)** | **C-reactive protein subgroup (n = 391)** |
| --- | --- | --- |
| PNI, AUC | 0.549 | 0.547 |
| Serum albumin, AUC | 0.557 | 0.555 |
| RDW, AUC | 0.597 | 0.594 |
| MELD, AUC | 0.674 | 0.672 |
| PNI, adjusted OR per SD | 0.928 (p = 0.38) | 0.935 (p = 0.44) |
| ΔC, MELD + PNI versus MELD | +0.005 (p = 0.48) | +0.006 (p = 0.44) |

**Panel C. Discrimination of in-hospital mortality**

| **Marker or model** | **n** | **AUC (95% CI)** | **ΔC versus MELD (p)** |
| --- | --- | --- | --- |
| PNI | 391 | 0.547 (0.493–0.601) | — |
| Serum albumin | 391 | 0.555 (0.501–0.609) | — |
| RDW | 391 | 0.594 (0.540–0.648) | — |
| C-reactive protein | 391 | 0.608 (0.553–0.663) | — |
| Procalcitonin | 308 | 0.587 (0.523–0.651) | — |
| MELD | 391 | 0.672 (0.621–0.723) | reference |
| MELD + C-reactive protein | 391 | 0.681 (0.631–0.731) | +0.009 (0.28) |
| MELD + PNI | 391 | 0.678 (0.627–0.729) | +0.006 (0.44) |
| MELD (procalcitonin subgroup) | 308 | 0.668 (0.600–0.736) | reference |
| MELD + procalcitonin | 308 | 0.676 (0.608–0.744) | +0.008 (0.36) |

**Panel D. Spearman correlations with the inflammatory markers**

|  | **C-reactive protein (n = 391)** | **Procalcitonin (n = 308)** |
| --- | --- | --- |
| Serum albumin | −0.371 | −0.289 |
| Lymphocyte count | −0.142 | −0.134 |
| PNI | −0.336 | −0.261 |
| MELD | +0.228 | +0.264 |
| RDW | +0.247 | +0.178 |
| C-reactive protein | — | +0.536 |

**Panel E1. Sequential adjustment of the PNI and serum albumin (odds ratios per standard deviation for in-hospital mortality)**

| **Model** | **PNI** | **Serum albumin** |
| --- | --- | --- |
| Unadjusted | 0.875 (0.741–1.033), p = 0.115 | 0.845 (0.715–0.999), p = 0.048 |
| + MELD, age, APACHE II | 0.935 (0.788–1.109), p = 0.44 | 0.903 (0.757–1.077), p = 0.25 |
| + C-reactive protein (untransformed) | 0.972 (0.814–1.160), p = 0.75 | 0.949 (0.790–1.141), p = 0.58 |
| + log C-reactive protein | 0.981 (0.820–1.173), p = 0.83 | 0.961 (0.799–1.156), p = 0.67 |
| + procalcitonin (untransformed), n = 308 | 0.953 (0.789–1.151), p = 0.62 | — |
| + log procalcitonin, n = 308 | 0.960 (0.794–1.161), p = 0.68 | — |

**Panel E2. The inflammatory markers themselves (odds ratios per standard deviation for in-hospital mortality)**

| **Marker (scale)** | **n** | **Unadjusted** | **Adjusted for MELD, age and APACHE II** |
| --- | --- | --- | --- |
| C-reactive protein (untransformed) | 391 | 1.365 (1.143–1.630), p < 0.001 | 1.197 (0.992–1.444), p = 0.061 |
| C-reactive protein (log) | 391 | — | 1.234 (1.014–1.502), p = 0.036 |
| Procalcitonin (untransformed) | 308 | — | 1.175 (0.954–1.447), p = 0.13 |
| Procalcitonin (log) | 308 | — | 1.208 (0.978–1.492), p = 0.079 |

*C-reactive protein and procalcitonin were taken as the first available value within 72 hours of ICU admission and were not truncated. Correlations are Spearman coefficients; all reported coefficients had p < 0.01. Dashes in Panel E denote combinations not estimated (the untransformed and log scales of the same marker are alternatives, not sequential adjustments). Odds ratios are per one–standard-deviation increment; models labelled log use the natural logarithm of the inflammatory marker, which is right-skewed. All models in Panels E1 and E2 are fitted in the C-reactive protein subgroup (n = 391) except those involving procalcitonin (n = 308). The C-reactive protein subgroup reproduced the full-cohort estimates (Panel B), indicating that measurement of C-reactive protein was not selective with respect to the associations under study. Neither inflammatory marker was measured at a coverage permitting analysis in the public cohorts: C-reactive protein was recorded within the ascertainment window for 347 of 7,786 MIMIC-IV patients with liver disease (4.5%) and for a comparable minority of eICU-CRD admissions, and procalcitonin and interleukin-6 are not systematically available in either database. Abbreviations as in the main text.*

The MELD score and the PNI are not ascertained by an identical rule. In MIMIC-IV the MELD components are the most abnormal values recorded on the first ICU day, whereas the PNI uses the first value available in the ascertainment window. Because the central comparison of this study is between these two scores, MELD was recomputed from the first value of each component in the same window used for the PNI.

**Supplementary Table S14. Discrimination of MELD under the ascertainment rule used for the PNI.**

| **Cohort** | **n** | **MELD, primary derivation (95% CI)** | **MELD, first value in the PNI window (95% CI)** | **PNI (95% CI)** | **ΔAUC, recomputed MELD − PNI (p)** |
| --- | --- | --- | --- | --- | --- |
| MIMIC-IV (28-day mortality) | 3,569 | 0.697 (0.679–0.715) | 0.685 (0.667–0.704) | 0.559 (0.539–0.579) | 0.126 (4.9×10⁻¹⁹) |

*This analysis is carried out in MIMIC-IV, the cohort in which the rule used to ascertain the MELD components is documented independently of this study and can therefore be contrasted directly with the window-based recalculation: MELD is taken from the corresponding derived concept of the MIMIC Code Repository, which applies the MELD formula to the most abnormal value of each component recorded on the first ICU day. The recomputed score applies the same formula and the same bounds to the first available value of each component between 6 hours before and 72 hours after ICU admission, the window used for the PNI. The two scores correlated at r = 0.93. The comparison is restricted to the 3,569 of 3,653 analysed patients in whom all three components were available within that window, a different denominator from the 3,516 patients for whom MELD 3.0 could be computed (Supplementary Table S10), which uses the first-ICU-day components; the PNI estimate shown is for the 3,569-patient subset. The ascertainment rule accounts for a difference of about 0.012 AUC units, an order of magnitude smaller than the difference between MELD and the PNI, so it does not explain the superiority of MELD. In eICU-CRD and the institutional cohort MELD is computed from the first value of each component in the same window as the PNI, so the two scores are ascertained by an identical rule there and this analysis does not arise.*

***Reporting checklists***

This observational study is reported in accordance with the STROBE statement, and the prognostic-marker analyses follow the relevant TRIPOD recommendations. The completed checklists are provided as separate files at submission and are not reproduced here.
